# Longitudinal Cervical Length Measurements and Spontaneous Preterm Birth in Singleton and Twin Pregnancies

**DOI:** 10.1001/jamanetworkopen.2024.4592

**Published:** 2024-04-11

**Authors:** Tianchen Wu, Shuang Li, Xiaoli Gong, Jiaxin Li, Xuening Li, Yujia Zhai, Jiaqi Huang, Xiaona Li, Luyao Li, Jing Yang, Xueju Wang, Huifeng Shi, Pengbo Yuan, Yangyu Zhao, Yuan Wei

**Affiliations:** 1 Department of Obstetrics and Gynecology, Peking University Third Hospital, Beijing, China; 2 National Centre for Healthcare Quality Management in Obstetrics, Beijing, China; 3 National Clinical Research Center for Obstetrics and Gynecology, Peking University Third Hospital, Beijing, China; 4 State Key Laboratory of Female Fertility Promotion, Peking University Third Hospital, Beijing, China; 5 Department of Pharmacy, Peking University Third Hospital, Beijing, China

## Abstract

**Importance:**

Changes in cervical length in twin pregnancies exhibit various patterns, but it is unclear whether the mechanism underlying spontaneous preterm birth (sPTB) is consistent. The existence of detailed phenomena in singleton pregnancies is also unclear.

**Objectives:**

To explore the different patterns in cervical length trajectories in singleton and twin pregnancies and to analyze whether the immunological mechanisms of sPTB are consistent among these cervical length patterns.

**Design, Setting, and Participants:**

This cohort study recruited pregnant individuals who received antenatal care and delivered at Peking University Third Hospital in Beijing, China, between January 1, 2014, and December 31, 2022. Individuals with singleton and twin pregnancies were included.

**Exposures:**

Cervical length measurements and white blood cell (WBC) indicators.

**Main Outcomes and Measures:**

The primary outcome was sPTB. Longitudinal trajectory cluster analysis was used to identify patterns of changes in cervical length in singleton and twin pregnancies. A random-effects model with cubic spline was used to fit and compare the longitudinal trajectory of WBC indicators among early preterm birth, moderate to late preterm birth, and term birth.

**Results:**

A total of 43 559 pregnant individuals were included; of these, 41 706 had singleton pregnancies (mean [SD)] maternal age, 33.0 [4.0] years) and 1853 had twin pregnancies (mean [SD] maternal age, 33.3 [3.6] years). Two distinct patterns of cervical length changes were observed in both singleton and twin pregnancies: shortened (21 366 singletons and 546 twins) and stable (20 340 singletons and 1307 twins). In singleton pregnancies, WBC count was associated with early sPTB in individuals with both shortened cervix (odds ratio [OR], 1.35; 95% CI, 1.00-1.82) and stable cervix (OR, 1.64; 95% CI, 1.07-2.50). However, for twin pregnancies, the association of WBC count (OR, 3.13; 95% CI, 1.58-6.18) with the risk of early sPTB was observed only in individuals with a shortened cervix.

**Conclusions and Relevance:**

This study identified 2 distinct cervical length patterns: shortened and stable. These patterns revealed 2 preterm birth mechanisms in twin pregnancies, with the immunopathogenesis of sPTB found only in the shortened cervix pattern; in singleton pregnancies, maternal immune response was associated with a higher risk of sPTB regardless of a shortened or stable cervix.

## Key Points

**Question:**

What disparities in the pathogenesis of spontaneous preterm birth (sPTB) exist among pregnant individuals with varying patterns of cervical length changes?

**Findings:**

In this cohort study of 41 706 participants with singleton pregnancies and 1853 participants with twin pregnancies, 2 distinct patterns of change in cervical length were observed among twin pregnancies: stable and shortened. For twin pregnancies, immune factors were associated only with sPTB in the shortened pattern; no association was observed in the stable pattern.

**Meaning:**

Findings of this study suggest that the mechanisms of sPTB in twin pregnancies are not entirely similar to those in singleton pregnancies; in particular, for twin pregnancies with a stable cervix, the sPTB is unrelated to immunological factors.

## Teaser

This cohort study examines cervical length trajectories and their association with risk of spontaneous preterm birth in individuals with singleton and twin pregnancies.

## Introduction

Preterm birth is the leading cause of morbidity and mortality for neonates and children younger than 5 years^1^ and is associated with increased risk of early childhood growth and developmental delays.^2^ In 2020, approximately 13.4 million infants worldwide were born prematurely, and from 2010 to 2020, the global preterm birth rate remained stable, with approximately 15% of births occurring before 32 weeks of gestation.^3^ In China, the prevalence of preterm birth was 5.7% for singleton pregnancies and 52.7% for multiple pregnancies in 2018.^4^ Among the clinical etiologies of preterm birth, spontaneous preterm birth (sPTB) accounts for two-thirds of cases.^5^ However, the mechanisms of sPTB, particularly in twin and multiple pregnancies, are multifactorial and still need to be elucidated.

Preterm labor syndrome is a heterogeneous condition with a common end point of delivery earlier than 37 weeks.^6^ Immunity is the main factor in sPTB, with maternal and/or fetal anatomy, endocrine, physiological, biochemical, and clinical factors also playing potential roles.^7^ Cervical length shortening is one of the early manifestations of these factors associated with preterm birth.^8^

The release of cytokines and chemokines by the maternal-fetal interface tissue recruits immune cells from the peripheral blood to migrate to the pregnancy tissue; as the immune cells release more proinflammatory mediators, an inflammatory cascade reaction is triggered, ultimately playing a role in preterm birth.^9^ Several cytokines associated with sPTB have been identified from cervicovaginal secretions, peripheral blood, umbilical cord blood, amniotic fluid, and placenta. These cytokines can be proinflammatory, such as tumor necrosis factor, interleukin 6 (IL-6), IL-8, and IL-1α,^10,11,12,13^ and anti-inflammatory, such as IL-4, IL-10, and IL-37.^14,15^ However, these findings were all from studies of singleton pregnancies. In contrast, only IL-8 was observed to be associated with sPTB in twin pregnancies.^16,17,18^

Cervical length, as measured by transvaginal sonography, is the most commonly used indicator of sPTB. A cervical length of less than 25 mm in the second trimester is the established cutoff value for assessing the risk of preterm birth.^19^ Serial measurements of cervical length have demonstrated the potential to enhance the identification of individuals at risk of sPTB.^20,21^ In twin pregnancies, serial measurements have revealed distinct patterns in cervical length trajectories. Individuals with a consistently shortened cervix throughout pregnancy carry the highest risk of sPTB, exhibiting at least a 2-fold increased risk compared with those with a stable cervix.^22^ A shortened cervix not only represents an anatomical abnormality but also indicates an underlying immunoregulatory imbalance at the maternal-fetal interface.^23^ The decrease in cervical length in the second trimester is associated with imbalances in the reproductive tract microbiome,^24^ chorioamnionitis,^25^ and increased peripheral blood cytokine levels, such as IL-6 and IL-10.^26^

In addition to immunopathogenesis, sPTB may also result from nonimmunological factors, such as uterine overdistension,^27^ abnormal amniotic fluid, and cervical or hormonal disorders.^6^ Currently, there is a scarcity of studies addressing the clinical differentiation of whether sPTB is immune-related. The present cohort study had 2 main objectives: (1) to explore the different patterns in cervical length trajectories in singleton and twin pregnancies, and (2) to analyze whether the immunological mechanisms (measured by white blood cell [WBC] indicators in routine blood tests) of sPTB are consistent among these different cervical length clusters. To conduct the study, we used data from a large clinical cohort.

## Methods

### Design and Participants

For this retrospective cohort study, we enrolled pregnant individuals who received antenatal care from the first trimester and delivered at the Peking University Third Hospital in Beijing, China, between January 1, 2014, and December 31, 2022. Original data were extracted from the electronic medical record, picture archiving and communication system, and laboratory information system of the hospital information system. The Peking University Third Hospital Medical Science Research Ethics Committee approved this study and waived the informed consent requirement because retrospective data were used and obtaining consent from each participant was not feasible. We followed the Strengthening the Reporting of Observational Studies in Epidemiology (STROBE) reporting guideline.

**Figure 1.**
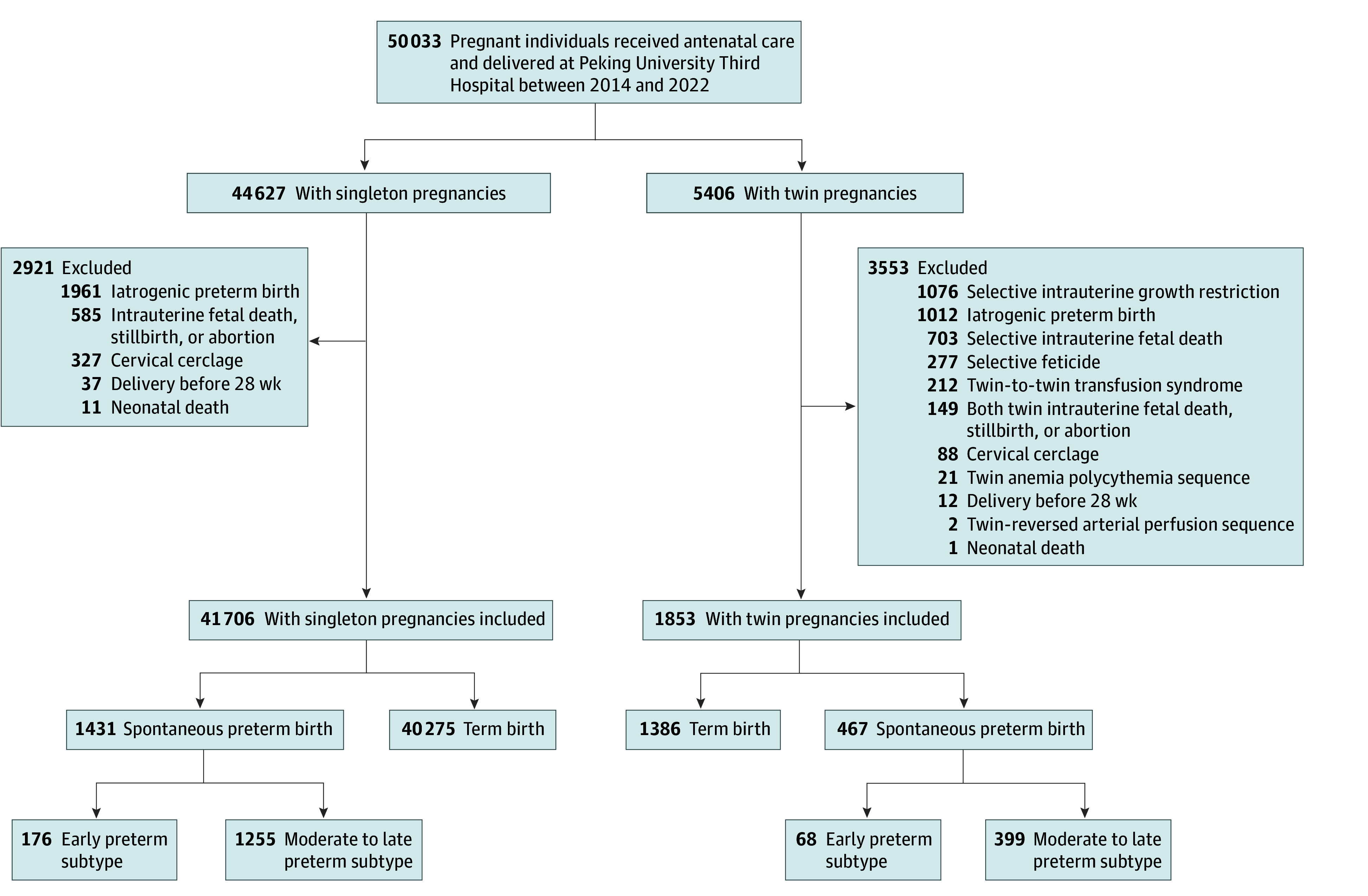
Flowchart of Participants Selection

Data from pregnant individuals with singleton and twin pregnancies were included and analyzed separately. Individuals with intrauterine death, stillbirth of 1 or both fetuses, abortion, iatrogenic premature birth, or cervical cerclage were excluded. Additionally, patients who delivered before 28 gestational weeks or experienced neonatal deaths were excluded. For twin pregnancies, cases of twin-to-twin transfusion syndrome, selective intrauterine growth restriction, twin reversed arterial perfusion sequence, twin anemia polycythemia sequence, genetic or structural anomalies, or selective fetal reduction were also excluded. The flowchart of participant selection is shown in Figure 1.

### Exposure Assessment

The primary exposures were cervical length measurements and laboratory tests. All cervical length measurements were performed by senior sonographers using transvaginal ultrasonography (Philips iU22 [Philips] or Voluson E8 [GE HealthCare]) with an ultrasound probe frequency of 4.0 to 8.0 MHz. The length between the cervical internal and external os was measured in the sagittal plane after the participant had emptied their bladder. Three repeated measurements were performed, and the minimum value was recorded. Hematological tests were conducted using automated hematological systems (Sysmex XN series).^28^ We collected test dates and WBC indicators from hematological tests on maternal peripheral blood, including total WBC, lymphocyte, neutrophil, monocyte, basophil, and eosinophil counts. All ultrasonographic measurement and laboratory test data were derived from routine antenatal care. The neutrophil to lymphocyte ratio (NLR) was calculated by dividing the neutrophil count by the lymphocyte count, and the lymphocyte to monocyte ratio (LMR) was calculated by dividing the lymphocyte count by the monocyte count. Each participant underwent a mean (SD) of 2.92 (1.07) cervical length measurements and 7.62 (2.06) hematological tests (eTables 1 and 2; eFigures 1 and 2 in Supplement 1).

### Outcome Definition

The primary outcome of this study was sPTB, defined as the onset of spontaneous labor or preterm premature rupture of membranes between 28 and 37 gestational weeks. Spontaneous preterm birth was further categorized as early preterm birth (delivery before 32 gestational weeks) and moderate to late preterm birth (delivery between 32 and 37 gestational weeks). The gestational age at delivery was obtained by calculating the interval between the delivery date and the last menstrual period date from clinical records.

We also collected neonatal outcomes, including birth weight, birth length, and admission to the neonatal intensive care unit. The prevalence rates of small for gestational age and large for gestational age for singleton^29^ and twin^30^ pregnancies were calculated using the Chinese population birth weight reference.

### Covariate Assessment

We collected maternal characteristics, including age, height, prepregnancy weight, gestational weight gain, parity, and conception mode. Additionally, we documented diagnoses of gestational complications, including gestational diabetes, hypertensive disorders of pregnancy, and preeclampsia. Prepregnancy body mass index (BMI; calculated as weight in kilograms divided by height in meters squared) was classified as follows: underweight (BMI <18.5), normal weight (BMI ≤18.5 to <24.0), overweight (BMI ≤24.0 to <28.0), and obesity (BMI ≥28.0).

### Statistical Analysis

We initially conducted longitudinal trajectory clustering on longitudinal repeated-measurement records of cervical length. The cluster was implemented using the clustra package, version 0.1.6, in R (R Project for Statistical Computing). The optimal number of clusters was identified by maximizing the mean silhouette value and adjusted Rand index.

Next, we described and compared maternal characteristics, gestational complications, and delivery outcomes between the different cervical length clusters. Between-group comparisons of continuous and categorical variables were performed using an unpaired, 2-tailed *t* test or χ^2^ test. Kaplan-Meier survival analysis was used to compare the gestational age at delivery between the clusters, and survival differences were ascertained by log-rank test. The random-effects model with natural cubic splines was used to fit the cervical length curve.

Then, we explored whether the immunopathogenesis of sPTB differed between the cervical length clusters. Participants within each cluster were initially subdivided into 3 subgroups: term birth, early preterm birth, and moderate to late preterm birth. Longitudinal curves of 8 WBC indicators were fitted in each subgroup using a random-effects model with natural cubic splines. These curves were compared among the 3 subgroups within each cluster using a likelihood ratio test. The between-group differences of 8 WBC indicator curves were quantified by calculating the *z* scores for early preterm birth and moderate to late preterm birth with reference to the term birth.

Furthermore, we divided the gestational age before 28 weeks into 7 periods at 4-week intervals, and we evaluated the association between 8 *z* score–standardized WBC indicators and sPTB during each period using unconditional logistic regression within a distinct cervical length cluster. Maternal characteristics and gestational complications were adjusted in the model. The interaction between WBC indicators and cervical length cluster was tested by including their interaction terms in the multivariable models.

All statistical analyses were performed separately for singleton and twin pregnancies. Statistical tests were 2-sided, with a significance level of α = .05. Analyses were conducted using SAS, version 9.4 (SAS Institute Inc), and R, version 4.3.1.

## Results

A total of 50 033 pregnant individuals were enrolled in this study, of whom 43 559 met the criteria and were included in the analysis. This cohort comprised 41 706 participants with singleton pregnancies and 1853 participants with twin pregnancies, with a mean (SD) age of 33.0 (4.0) years and 33.3 (3.6) years, respectively. Among these pregnancies, 1431 (3.4%) singletons and 467 (25.2%) twins were identified as sPTB (Table). Early preterm birth accounted for 12.3% of singletons and 14.6% of twins, whereas moderate to late preterm birth accounted for 87.7% of singletons and 85.4% of twins. The mean (SD) birth weight was 3312.0 (437.1) g for singleton pregnancies and 2582.9 (434.2) g for twin pregnancies. After delivery, 11.8% of singleton newborns and 23.2% of twin newborns were admitted to the neonatal intensive care unit (eTables 3 and 4 in Supplement 1).

**Table.** Maternal Characteristics of Singleton and Twin Pregnancies Stratified by Cervical Length Clustering Subgroup

Maternal characteristic	Singleton pregnancies, No. (%)			Twin pregnancies, No. (%)		
	All (n = 41 706)	Cervical length		All (n = 1853)	Cervical length	
		Stable (n = 20 340)	Shortened (n = 21 366)		Stable (n = 1307)	Shortened (n = 546)
Age, mean (SD), y	33.0 (4.0)	33.6 (4.0)	32.5 (3.9)	33.3 (3.6)	33.5 (3.7)	32.9 (3.5)
≤35	29 230 (70.1)	13 276 (65.3)	15 954 (74.7)	1266 (68.3)	862 (66.0)	404 (74.0)
>35	12 476 (29.9)	7064 (34.7)	5412 (25.3)	587 (31.7)	445 (34.1)	142 (26.0)
Height, mean (SD), cm	162.5 (5.2)	162.5 (5.3)	162.5 (5.0)	162.9 (5.1)	162.8 (5.1)	162.9 (5.2)
Prepregnancy weight, mean (SD), kg	58.5 (9.0)	59.8 (9.3)	57.3 (8.6)	59.7 (9.1)	60.1 (9.2)	58.8 (9.0)
Prepregnancy BMI, mean (SD)^a^	22.1 (3.2)	22.6 (3.3)	21.7 (3.0)	22.5 (3.2)	22.7 (3.2)	22.2 (3.3)
Underweight	3787 (9.1)	1311 (6.5)	2476 (11.6)	136 (7.3)	84 (6.4)	52 (9.5)
Normal weight	28 192 (67.6)	13 332 (65.6)	14 860 (69.6)	1202 (64.9)	841 (64.4)	361 (66.1)
Overweight	7459 (17.9)	4319 (21.2)	3140 (14.7)	398 (21.5)	295 (22.6)	103 (18.9)
Obesity	2268 (5.4)	1378 (6.8)	890 (4.2)	117 (6.3)	87 (6.7)	30 (5.5)
Gestational weight gain, mean (SD), kg	13.01 (4.4)	13.15 (4.5)	12.88 (4.3)	15.73 (5.2)	16.03 (5.2)	15.01 (5.1)
Parity						
Primipara	29 370 (70.4)	13 092 (64.4)	16 278 (76.2)	1544 (83.3)	1056 (80.8)	488 (89.4)
Multipara	12 336 (29.6)	7248 (35.6)	5088 (23.8)	309 (16.7)	251 (19.2)	58 (10.6)
Conception mode						
Spontaneous	37 230 (89.3)	18 066 (88.8)	19 164 (89.7)	743 (40.1)	571 (43.7)	172 (31.5)
ART	4476 (10.7)	2274 (11.2)	2202 (10.3)	1110 (59.9)	736 (56.3)	374 (68.5)
Gestational diabetes						
No	30 718 (73.7)	14 920 (73.4)	15 798 (73.9)	1325 (71.5)	959 (73.4)	366 (67.0)
Yes	10 988 (26.4)	5420 (26.7)	5568 (26.1)	528 (28.5)	348 (26.6)	180 (33.0)
Hypertensive disorders of pregnancy						
No	38 413 (92.1)	18 530 (91.1)	19 883 (93.1)	1643 (88.7)	1167 (89.3)	476 (87.2)
Yes	3293 (7.9)	1810 (8.9)	1483 (6.9)	210 (11.3)	140 (10.7)	70 (12.8)
Preeclampsia						
No	40 337 (96.7)	19 571 (96.2)	20 766 (97.2)	1726 (93.2)	1226 (93.8)	500 (91.6)
Yes	1369 (3.3)	769 (3.8)	600 (2.8)	127 (6.9)	81 (6.2)	46 (8.4)
Gestational age at delivery, mean (SD), wk	39.2 (1.3)	39.3 (1.2)	39.2 (1.4)	36.8 (2.0)	37.2 (1.7)	36.0 (2.3)
sPTB						
No	40 275 (96.6)	19 793 (97.3)	20 482 (95.9)	1386 (74.8)	1069 (81.8)	317 (58.1)
Yes	1431 (3.4)	547 (2.7)	884 (4.1)	467 (25.2)	238 (18.2)	229 (41.9)
Subtype of sPTB						
Moderate to late preterm birth	1255 (3.0)	490 (2.4)	765 (3.6)	399 (21.5)	204 (15.6)	195 (35.7)
Early preterm birth	176 (0.4)	57 (0.3)	119 (0.6)	68 (3.7)	34 (2.6)	34 (6.2)

Abbreviations: ART, assisted reproductive technology; BMI, body mass index (calculated as weight in kilograms divided by height in meters squared); sPTB, spontaneous preterm birth.

^a^ Underweight: BMI less than 18.5; normal weight: BMI 18.5 to less than 24.0; overweight: BMI greater than or equal to 24.0 to less than 28.0; obesity: BMI greater than or equal to 28.0.

### Longitudinal Clustering of Cervical Length Trajectories

**Figure 2.**
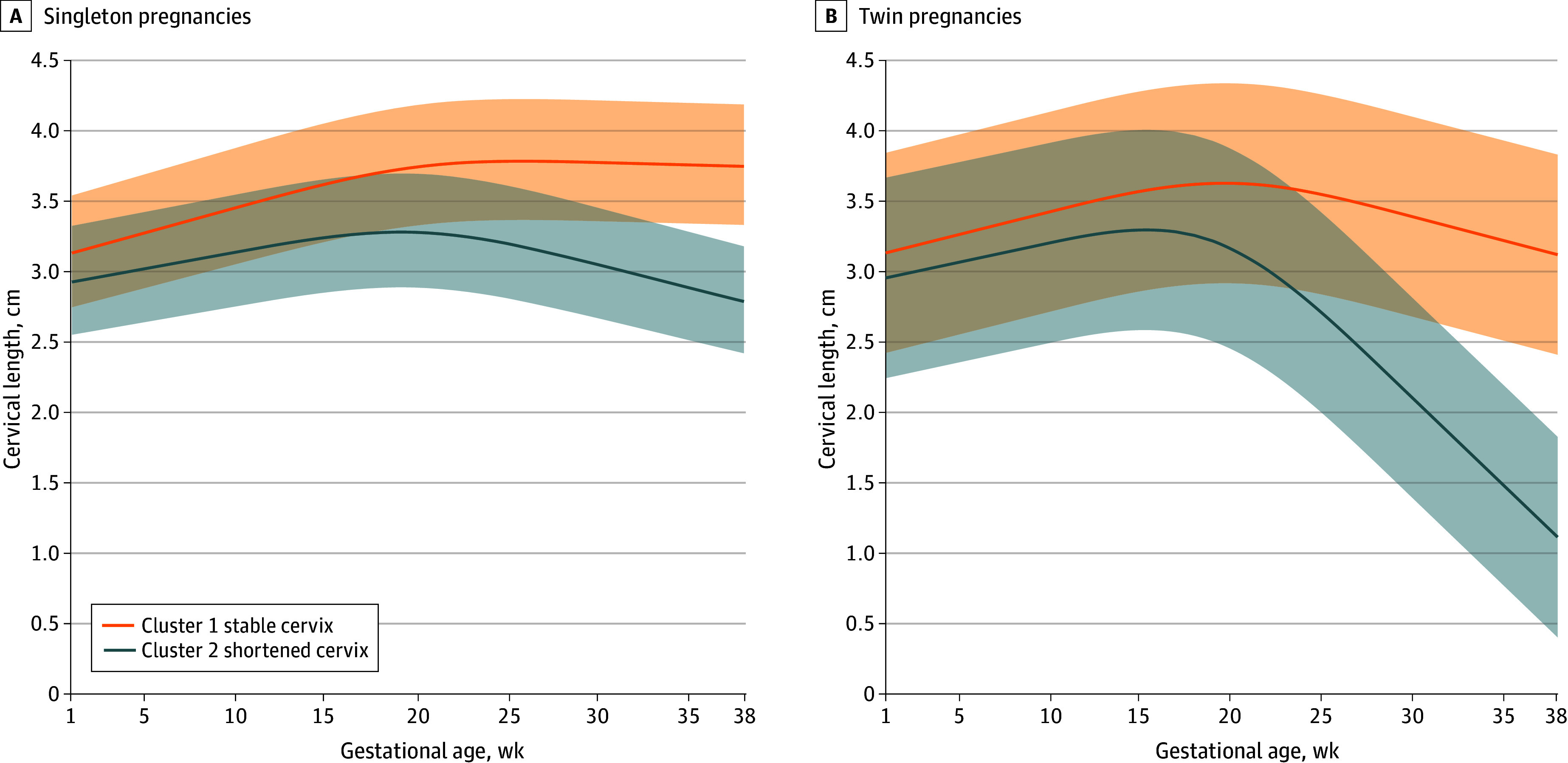
Two Longitudinal Trajectory Patterns of Cervical Length in Singleton and Twin Pregnancies The shaded areas represent 95% CIs.

The optimal number of clusters for longitudinal cervical length clustering in singleton and twin pregnancies was 2, with mean silhouette values of 0.68 and 0.76, respectively, reaching their peaks. The adjusted Rand index also reached its maximum value (eFigures 3 to 6 in Supplement 1). Consequently, both singleton and twin pregnancies exhibited 2 distinct patterns in cervical length trajectories (Figure 2). The first pattern reflected a stable cervix (20 340 singletons and 1307 twins), with a gradual decrease in cervical length throughout gestation. The median (IQR) changes in cervical length were 5.6 (4.3-6.8) mm for singleton pregnancies and 5.8 (2.1-9.4) mm for twin pregnancies. The second pattern was the shortened cervix (21 366 singletons and 546 twins), with cervical length reducing rapidly from the second trimester. The median (IQR) changes in cervical length after the second trimester were 6.9 (5.7-8.1) mm for singleton pregnancies and 24.3 (20.7-28.0) mm for twin pregnancies. Pregnant individuals with a shortened cervix had a higher prevalence of sPTB compared with those with a stable cervix in both singleton (4.1% vs 2.7%) and twin (18.2% vs 41.9%) pregnancies (Table). Survival analysis further revealed that pregnant individuals with a shortened cervix delivered earlier than those with a stable cervix (eFigure 7 in Supplement 1).

### Association of WBC Indicators With sPTB Stratified by Cervical Length Clusters

**Figure 3.**
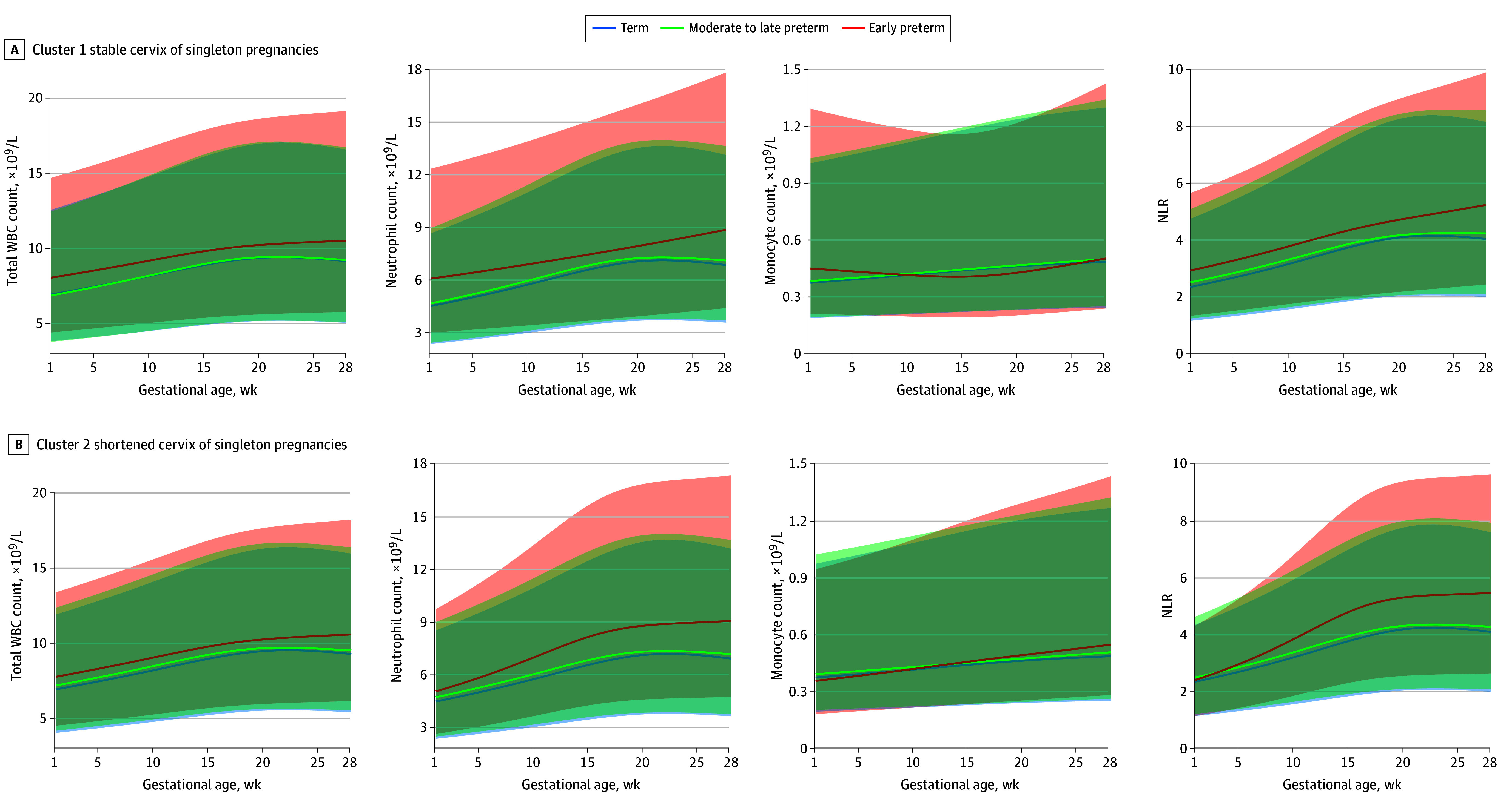
Gestational Age–Specific Trajectories for White Blood Cell (WBC) Indicators Stratified by Cervical Length Clusters in Singleton Pregnancies The shaded areas represent 95% CIs. NLR indicates neutrophil to lymphocyte ratio; WBC, white blood cell.

**Figure 4.**
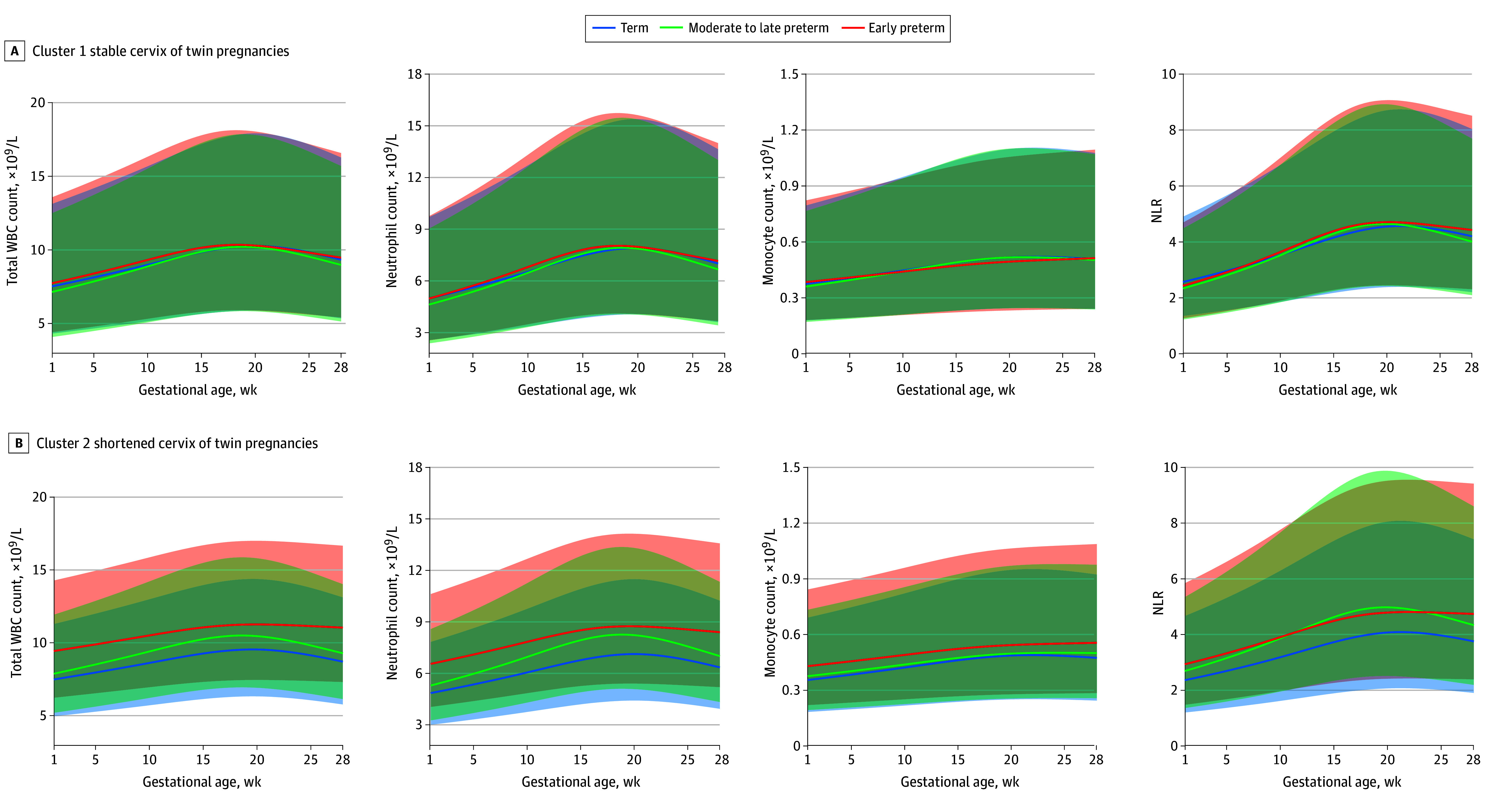
Gestational Age–Specific Trajectories for White Blood Cell (WBC) Indicators Stratified by Cervical Length Clusters in Twin Pregnancies The shaded areas represent 95% CIs. NLR indicates neutrophil to lymphocyte ratio; WBC, white blood cell.

In singleton pregnancies compared with the term birth subgroup, pregnant individuals with a shortened or stable cervix in the early preterm birth subgroup exhibited a higher total WBC count, neutrophil count, and NLR, whereas LMR was lower (Figure 3; eFigure 8 and eTable 5 in Supplement 1). However, in twin pregnancies, the increase in total WBC count, neutrophil count, monocyte count, and NLR as well as the decrease in LMR in the early preterm birth subgroup were observed only in individuals with a shortened cervix (Figure 4; eFigure 9 and eTable 5 in Supplement 1). The differences in 8 WBC indicators between early preterm birth, moderate to late preterm birth, and term birth subgroups were quantified using *z* scores, demonstrating similar findings (eFigures 10 to 13 in Supplement 1).

Furthermore, we quantified the association between WBC indicators and sPTB risk using logistic models. In singleton pregnancies, both WBC count (odds ratio [OR], 1.64 [95% CI, 1.07-2.50] for stable cervix cluster; OR, 1.35 [95% CI, 1.00-1.82] for shortened cervix cluster at 13-16 weeks) and neutrophil count (OR, 1.64 [95% CI, 1.07-2.50] for stable cervix cluster; OR, 1.35 [95% CI, 1.00-1.82] for shortened cervix cluster at 13-16 weeks) were associated with the risk of early preterm birth since 13 gestational weeks (eFigure 14 and eTable 6 in Supplement 1). In contrast, for twin pregnancies, the association of WBC count (OR, 3.13; 95% CI, 1.58-6.18) and neutrophil count (OR, 3.57; 95% CI, 1.73-7.36) with the risk of early preterm birth was observed only within the shortened cervix cluster since 13 gestational weeks. However, the monocyte count (OR, 1.50; 95% CI, 1.14-1.98) was associated with early preterm birth at the last period of the second trimester (25-28 gestational weeks) in the shortened cervix cluster of twin pregnancies (eFigure 15 and eTable 6 in Supplement 1).

## Discussion

In this cohort study, we observed 2 distinct patterns of cervical length trajectories: shortened and stable cervix clusters. These patterns revealed 2 different mechanisms of sPTB in twin pregnancies, with one related to the immune response and the other unrelated to the immune response. In twin pregnancies of participants with shortened cervix, immune activation was associated with a higher risk of sPTB, including increased total WBC count, neutrophil count, monocyte count, and NLR. In the stable cervix cluster of twin pregnancies, similar associations were not found. Conversely, in singleton pregnancies, there was an association between immune activation and sPTB in both the shortened and stable cervix clusters.

Although several studies have investigated the predictive value of serial cervical length monitoring for preterm birth,^20,31^ studies focusing on the association between patterns of cervical length change and preterm birth are still limited. Melamed et al^22^ identified 4 patterns of changes in cervical length in twin pregnancies, including stable cervix, early rapid shortening, late shortening, and early shortening with plateau, and found that the 3 shortened cervix subgroups had a 2-fold higher risk of preterm birth compared with the stable cervix subgroup. However, further research is needed to ascertain whether there are differences in the pathogenesis of sPTB among these cervical patterns in twin pregnancies.

In the present study, we optimized the classification into 2 categories and obtained similar results, with the risk of sPTB being 2-fold higher in the shortened cervix cluster than the stable cervix cluster in both singleton and twin pregnancies. Notably, the median difference in cervical length between the 2 clusters in singleton pregnancies never exceeded 1 cm, whereas the difference was substantial in twin pregnancies, with a median difference of more than 2 cm. This finding may suggest that individuals with a shortened or stable cervix share a similar pathogenesis of sPTB in singleton pregnancies but not in twin pregnancies.

Pregnancy is accompanied by precise immune regulation,^32^ the onset of labor involves serial programmed immune responses, and abnormal responses can increase the risk of sPTB.^33^ Clinical studies have reported the association of increased WBC count,^34,35,36,37^ neutrophil count,^35,38^ lymphocyte count,^35,37^ monocyte count,^37^ and NLR^39,40^ with an elevated risk of preterm birth. Therefore, in the present study, we also used WBC indicators from routine blood tests as markers of maternal inflammation. Consistent with these findings, this study found a similar association between increased WBC counts, neutrophil counts, and NLR and a higher risk of sPTB in singleton pregnancies regardless of whether the cervix was shortened or stable. However, these associations were observed only in twin pregnancies with a shortened cervix. The increase in WBC indicators in peripheral blood indicated heightened maternal immune response, and their association with preterm birth signified the involvement of immunopathogenesis in the development of sPTB.^9,33,41^ Nevertheless, in twin pregnancies with a stable cervix, WBC indicators were not associated with sPTB. This observation suggests that immune factors are not the primary mechanism underlying sPTB in these cases, although the exact mechanism requires further exploration.

### Limitations

This study has some limitations. First, the analysis of the pathogenesis of preterm birth was solely based on clinical data and the detection of maternal peripheral blood plasma. Future studies are necessary to confirm and validate these findings at the maternal-fetal interface tissue level. Second, this study did not include extremely preterm births delivered before 28 gestational weeks because of their low prevalence, which posed challenges in obtaining reliable analytical results. Third, the study could not establish a causal association between changes in cervical length and inflammation, as changes were measured concurrently during pregnancy. Fourth, multicenter clinical studies are warranted to assess the generalizability and consistency of the results across diverse populations.

## Conclusions

In this cohort study of singleton and twin pregnancies, 2 distinct patterns of changes in cervical length emerged: shortened cervix and stable cervix. By clustering the cervical length trajectories, we observed 2 mechanisms of sPTB in twin pregnancies. Immunopathogenesis was found only in the shortened cervic pattern, whereas the sPTB in the stable cervix pattern was unrelated to the maternal immune response. However, in singleton pregnancies, maternal immune response was associated with a higher risk of sPTB regardless of a shortened or stable cervix.

## Notes

Supplement 1. **eTable 1.** The Distribution of the Number of Cervical Length Examinations Throughout the Entire Pregnancy for Each Pregnant Woman **eFigure 1.** The Distribution of the Number of Cervical Length Examinations Throughout the Entire Pregnancy for Each Pregnant Woman **eTable 2.** The Distribution of the Number of Blood Routine Examinations (White Blood Cell Indicators) Throughout the Entire Pregnancy for Each Pregnant Woman **eFigure 2.** The Distribution of the Number of Blood Routine Examinations (White Blood Cell Indicators) Throughout the Entire Pregnancy for Each Pregnant Woman **eTable 3.** Neonatal Outcomes of Singleton Pregnancies **eTable 4.** Neonatal Outcomes of Twin Pregnancies **eFigure 3.** Adjusted Rand Index of Longitudinal Clustering of Cervical Length in Singleton Pregnancies **eFigure 4.** Average Silhouette Value of Longitudinal Clustering of Cervical Length in Singleton Pregnancies **eFigure 5.** Adjusted Rand Index of Longitudinal Clustering of Cervical Length in Twin Pregnancies **eFigure 6.** Average Silhouette Value of Longitudinal Clustering of Cervical Length in Twin Pregnancies **eFigure 7.** Survival Analysis of Gestational Age at Delivery Stratified by Cervical Length Clusters in Singleton and Twin Pregnancies **eFigure 8.** Gestational Age-Specific Trajectories for Lymphocytes, Basophils, Eosinophils, Lymphocyte-to-Monocyte Ratio Stratified by Cervical Length Clusters in Singleton Pregnancies **eFigure 9.** Gestational Age-Specific Trajectories for Lymphocytes, Basophils, Eosinophils, Lymphocyte-to-Monocyte Ratio Stratified by Cervical Length Clusters in Twin Pregnancies **eFigure 10.** Gestational Age-Specific Z-Score for White Blood Cell Indicators in Singleton Pregnancies With Stable Cervix With Reference to Term Birth **eFigure 11.** Gestational Age-Specific Z-Score for White Blood Cell Indicators in Singleton Pregnancies With Shortened Cervix With Reference to Term Birth **eFigure 12.** Gestational Age-Specific Z-Score for White Blood Cell Indicators in Twin Pregnancies With Stable Cervix With Reference to Term Birth **eFigure 13.** Gestational Age-Specific Z-Score for White Blood Cell Indicators in Twin Pregnancies With Shortened Cervix With Reference to Term Birth **eFigure 14.** The Relationship Between White Blood Cell Indicators and Spontaneous Preterm Birth Stratified by Cervical Length Clusters in Singleton Pregnancies **eFigure 15.** The Relationship Between White Blood Cell Indicators and Spontaneous Preterm Birth Stratified by Cervical Length Clusters in Twin Pregnancies **eTable 5.** Likelihood Ratio Test for Between-Group Comparison of White Blood Cell Indicators by Gestational Week of Delivery **eTable 6.** Estimating the Association Between White Blood Cell Indicators and Spontaneous Preterm Birth Using Logistic Regression With Reference to Term Birth

Supplement 2.
Data Sharing Statement
