# Supplementary material for: Longitudinal Cervical Length Measurements and Spontaneous Preterm Birth in Singleton and Twin Pregnancies
Source: JAMA Netw Open. 2024 Apr 11;7(4):e244592. doi: 10.1001/jamanetworkopen.2024.4592 (PMC11009824; doi:10.1001/jamanetworkopen.2024.4592)
Supplement: Supplement 1. — eTable 1. The Distribution of the Number of Cervical Length Examinations Throughout the Entire Pregnancy for Each Pregnant Woman eFigure 1. The Distribution of the Number of Cervical Length Examinations Throughout the Entire Pregnancy for Each Pregnant Woman eTable 2. The Distribution of the Number of Blood Routine Examinations (White Blood Cell Indicators) Throughout the Entire Pregnancy for Each Pregnant Woman eFigure 2. The Distribution of the Number of Blood Routine Examinations (White Blood Cell Indicators) Throughout the Entire Pregnancy for Each Pregnant Woman eTable 3. Neonatal Outcomes of Singleton Pregnancies eTable 4. Neonatal Outcomes of Twin Pregnancies eFigure 3. Adjusted Rand Index of Longitudinal Clustering of Cervical Length in Singleton Pregnancies eFigure 4. Average Silhouette Value of Longitudinal Clustering of Cervical Length in Singleton Pregnancies eFigure 5. Adjusted Rand Index of Longitudinal Clustering of Cervical Length in Twin Pregnancies eFigure 6. Average Silhouette Value of Longitudinal Clustering of Cervical Length in Twin Pregnancies eFigure 7. Survival Analysis of Gestational Age at Delivery Stratified by Cervical Length Clusters in Singleton and Twin Pregnancies eFigure 8. Gestational Age-Specific Trajectories for Lymphocytes, Basophils, Eosinophils, Lymphocyte-to-Monocyte Ratio Stratified by Cervical Length Clusters in Singleton Pregnancies eFigure 9. Gestational Age-Specific Trajectories for Lymphocytes, Basophils, Eosinophils, Lymphocyte-to-Monocyte Ratio Stratified by Cervical Length Clusters in Twin Pregnancies eFigure 10. Gestational Age-Specific Z-Score for White Blood Cell Indicators in Singleton Pregnancies With Stable Cervix With Reference to Term Birth eFigure 11. Gestational Age-Specific Z-Score for White Blood Cell Indicators in Singleton Pregnancies With Shortened Cervix With Reference to Term Birth eFigure 12. Gestational Age-Specific Z-Score for White Blood Cell Indicators in Twin Pregnancies With Stable Cervix With [file jamanetwopen-e244592-s001.pdf]

Supplemental Online Content

Wu T, Li S, Gong X, et al. Cervical length measurements and spontaneous preterm birth in singleton and twin pregnancies. *JAMA Netw Open*. 2024;7(4):e244592. doi:10.1001/jamanetworkopen.2024.4592

eTable 1. The Distribution of the Number of Cervical Length Examinations Throughout the Entire Pregnancy for Each Pregnant Woman

eFigure 1. The Distribution of the Number of Cervical Length Examinations Throughout the Entire Pregnancy for Each Pregnant Woman

eTable 2. The Distribution of the Number of Blood Routine Examinations (White Blood Cell Indicators) Throughout the Entire Pregnancy for Each Pregnant Woman

eFigure 2. The Distribution of the Number of Blood Routine Examinations (White Blood Cell Indicators) Throughout the Entire Pregnancy for Each Pregnant Woman

eTable 3. Neonatal Outcomes of Singleton Pregnancies

eTable 4. Neonatal Outcomes of Twin Pregnancies

eFigure 3. Adjusted Rand Index of Longitudinal Clustering of Cervical Length in Singleton Pregnancies

eFigure 4. Average Silhouette Value of Longitudinal Clustering of Cervical Length in Singleton Pregnancies

eFigure 5. Adjusted Rand Index of Longitudinal Clustering of Cervical Length in Twin Pregnancies

eFigure 6. Average Silhouette Value of Longitudinal Clustering of Cervical Length in Twin Pregnancies

eFigure 7. Survival Analysis of Gestational Age at Delivery Stratified by Cervical Length Clusters in Singleton and Twin Pregnancies

eFigure 8. Gestational Age-Specific Trajectories for Lymphocytes, Basophils, Eosinophils, Lymphocyte-to-Monocyte Ratio Stratified by Cervical Length Clusters in Singleton Pregnancies

© 2024 Wu T et al. *JAMA Network Open*

eFigure 9. Gestational Age-Specific Trajectories for Lymphocytes, Basophils, Eosinophils, Lymphocyte-to-Monocyte Ratio Stratified by Cervical Length Clusters in Twin Pregnancies

eFigure 10. Gestational Age-Specific Z-Score for White Blood Cell Indicators in Singleton Pregnancies With Stable Cervix With Reference to Term Birth

eFigure 11. Gestational Age-Specific Z-Score for White Blood Cell Indicators in Singleton Pregnancies With Shortened Cervix With Reference to Term Birth

eFigure 12. Gestational Age-Specific Z-Score for White Blood Cell Indicators in Twin Pregnancies With Stable Cervix With Reference to Term Birth

eFigure 13. Gestational Age-Specific Z-Score for White Blood Cell Indicators in Twin Pregnancies With Shortened Cervix With Reference to Term Birth

eFigure 14. The Relationship Between White Blood Cell Indicators and Spontaneous Preterm Birth Stratified by Cervical Length Clusters in Singleton Pregnancies

eFigure 15. The Relationship Between White Blood Cell Indicators and Spontaneous Preterm Birth Stratified by Cervical Length Clusters in Twin Pregnancies

eTable 5. Likelihood Ratio Test for Between-Group Comparison of White Blood Cell Indicators by Gestational Week of Delivery

eTable 6. Estimating the Association Between White Blood Cell Indicators and Spontaneous Preterm Birth Using Logistic Regression With Reference to Term Birth

This supplemental material has been provided by the authors to give readers additional information about their work.

eTable 1. The distribution of the number of cervical length examinations throughout the entire pregnancy for each pregnant woman

	The number of cervical length examinations			
	2	3	4	≥5
Singleton, n(%)	18209 (49.2)	9407 (25.4)	5104 (13.8)	4329 (11.7)
Twin, n(%)	337 (19.7)	304 (17.8)	333 (19.5)	735 (43.0)

© 2024 Wu T et al. *JAMA Network Open*

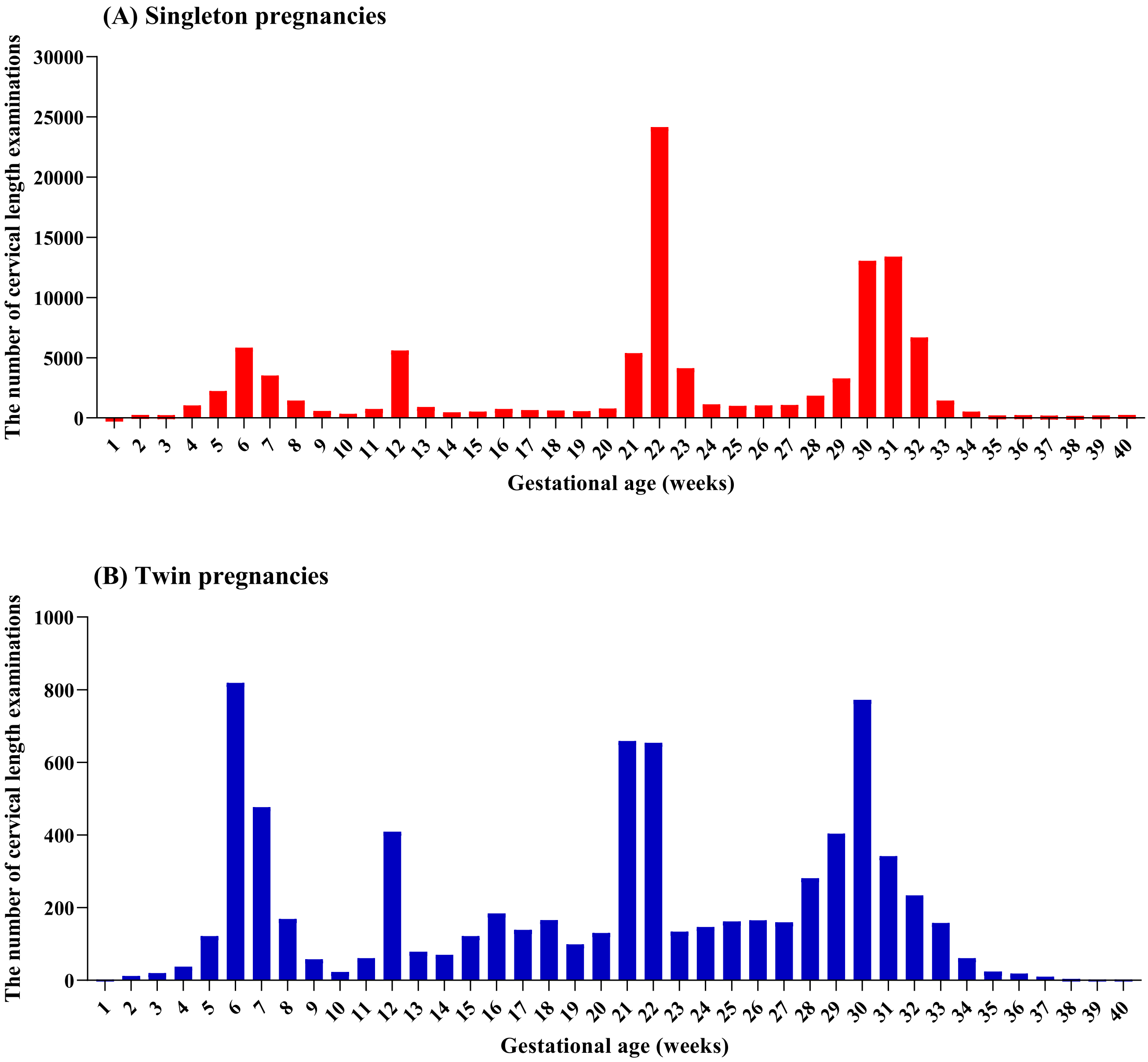

eFigure 1. The distribution of the number of cervical length examinations throughout the entire pregnancy for each pregnant woman

© 2024 Wu T et al. *JAMA Network Open*

eTable 2. The distribution of the number of blood routine examinations (white blood cell indicators) throughout the entire pregnancy for each pregnant woman

	The number of blood routine examinations (white blood cell indicators)										
	2	3	4	5	6	7	8	9	10	11	≥12
Singleton, n(%)	114 (0.3)	753 (1.8)	1051 (2.5)	2865 (6.9)	8056 (19.3)	9623 (23.1)	7571 (18.2)	4605 (11.1)	2725 (6.5)	1615 (3.9)	2692 (6.5)
Twin, n(%)	1 (0.1)	30 (1.6)	59 (3.2)	103 (5.6)	198 (10.7)	291 (15.7)	313 (16.9)	264 (14.3)	187 (10.1)	123 (6.6)	284 (15.3)

© 2024 Wu T et al. *JAMA Network Open*

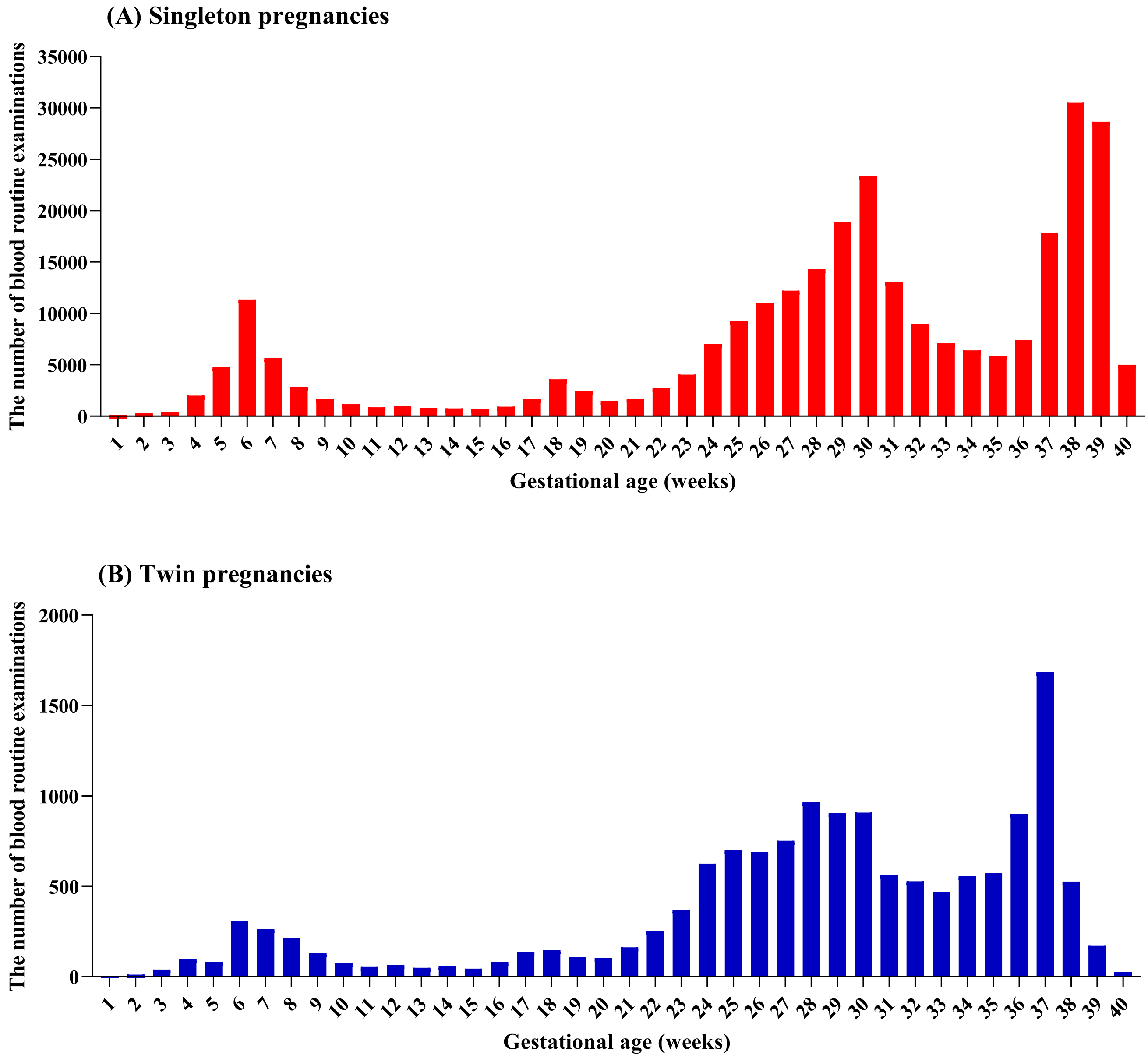

eFigure 2. The distribution of the number of blood routine examinations (white blood cell indicators) throughout the entire pregnancy for each pregnant woman

© 2024 Wu T et al. *JAMA Network Open*

eTable 3. Neonatal outcomes of singleton pregnancies

Neonatal Outcome	All (n=43487)	Cervical length	
		Stable (n=21400)	Shortened (n=22087)
Neonatal intensive care unit, n(%)			
No	36802 (88.2)	17974 (88.4)	18828 (88.1)
Yes	4904 (11.8)	2366 (11.6)	2538 (11.9)
Gender of the newborn, n(%)			
Boys	21579 (51.7)	10574 (52.0)	11005 (51.5)
Girls	20127 (48.3)	9766 (48.0)	10361 (48.5)
Birth weight, (g)	3312.0±437.1	3349.8±426.8	3276.0±443.7
Birth length, (cm)	49.5±2.1	49.7±2.0	49.3±2.2
Small for gestational age, n(%)			
No	39259 (94.1)	19310 (94.9)	19949 (93.4)
Yes	2447 (5.9)	1030 (5.1)	1417 (6.6)
Large for gestational age, n(%)			
No	35191 (84.4)	16791 (82.6)	18400 (86.1)
Yes	6515 (15.6)	3549 (17.5)	2966 (13.9)

© 2024 Wu T et al. *JAMA Network Open*

eTable 4. Neonatal outcomes of twin pregnancies

Neonatal Outcome	All (n=1953)	Cervical length	
		Stable (n=1369)	Shortened (n=584)
Neonatal intensive care unit, n(%)			
No	1423 (76.8)	1044 (79.9)	379 (69.4)
Yes	430 (23.2)	263 (20.1)	167 (30.6)
Gender of the newborn of fetus one, n(%)			
Boys	1017 (54.9)	723 (55.3)	294 (53.9)
Girls	836 (45.1)	584 (44.7)	252 (46.2)
Birth weight of fetus one, (g)	2582.9±434.2	2639.6±399.0	2447.2±482.6
Birth length of fetus one, (cm)	46.3±2.9	46.7±2.5	45.5±3.5
Small for gestational age of fetus one, n(%)			
No	1822 (98.3)	1284 (98.2)	538 (98.5)
Yes	31 (1.7)	23 (1.8)	8 (1.5)
Large for gestational age of fetus one, n(%)			
No	1503 (81.1)	1047 (80.1)	456 (83.5)
Yes	350 (18.9)	260 (19.9)	90 (16.5)
Gender of the newborn of fetus two, n(%)			
Boys	971 (52.4)	690 (52.8)	281 (51.5)
Girls	882 (47.6)	617 (47.2)	265 (48.5)
Birth weight of fetus two, (g)	2528.6±425.9	2585.3±396.1	2393.0±463.2
Birth length of fetus two, (cm)	46.2±2.8	46.6±2.6	45.4±3.0
Small for gestational age of fetus two, n(%)			
No	1782 (96.2)	1258 (96.3)	524 (96.0)
Yes	71 (3.8)	49 (3.8)	22 (4.0)
Large for gestational age of fetus two, n(%)			
No	1575 (85.0)	1103 (84.4)	472 (86.5)
Yes	278 (15.0)	204 (15.6)	74 (13.6)

© 2024 Wu T et al. *JAMA Network Open*

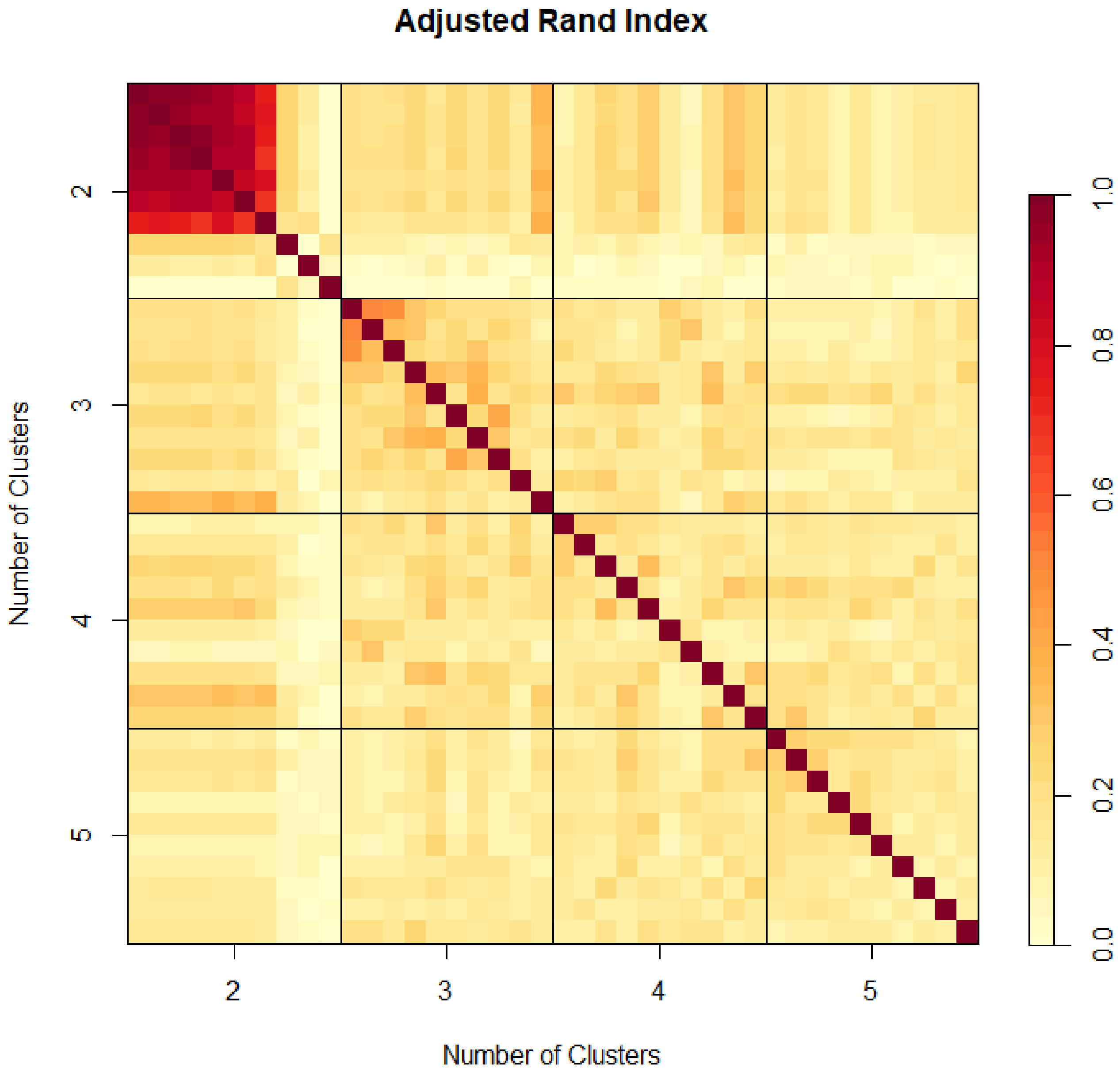

eFigure 3. Adjusted Rand index of longitudinal clustering of cervical length in singleton pregnancies

© 2024 Wu T et al. *JAMA Network Open*

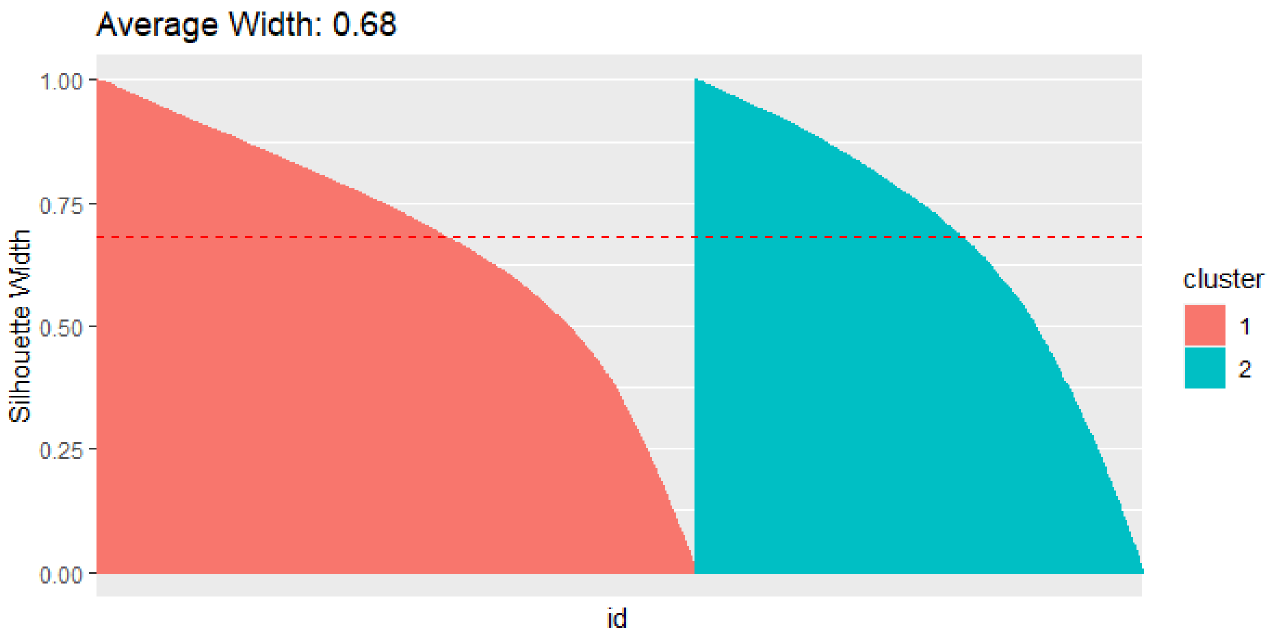

eFigure 4. Average silhouette value of longitudinal clustering of cervical length in singleton pregnancies

© 2024 Wu T et al. *JAMA Network Open*

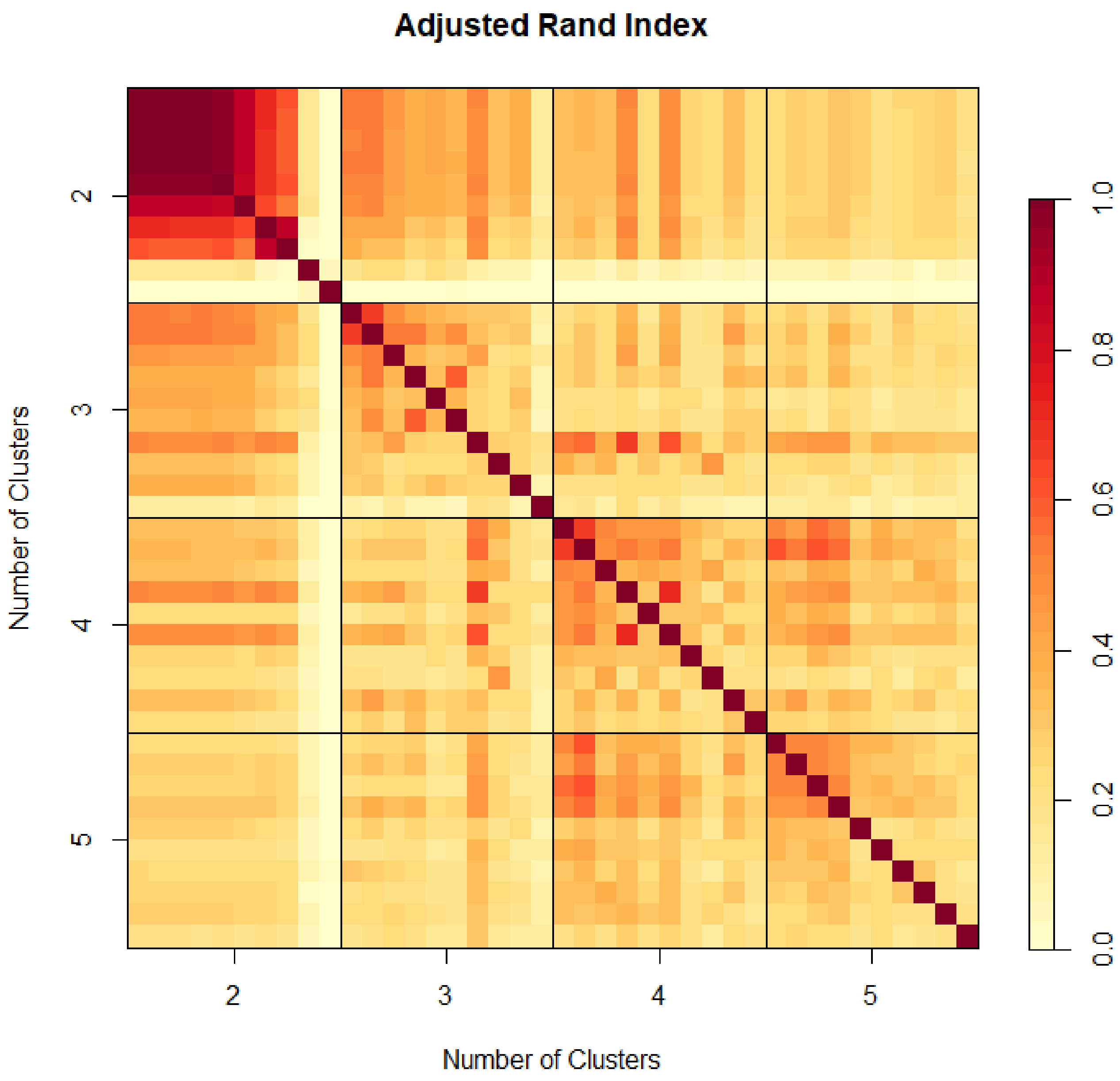

eFigure 5. Adjusted Rand index of longitudinal clustering of cervical length in twin pregnancies

© 2024 Wu T et al. *JAMA Network Open*

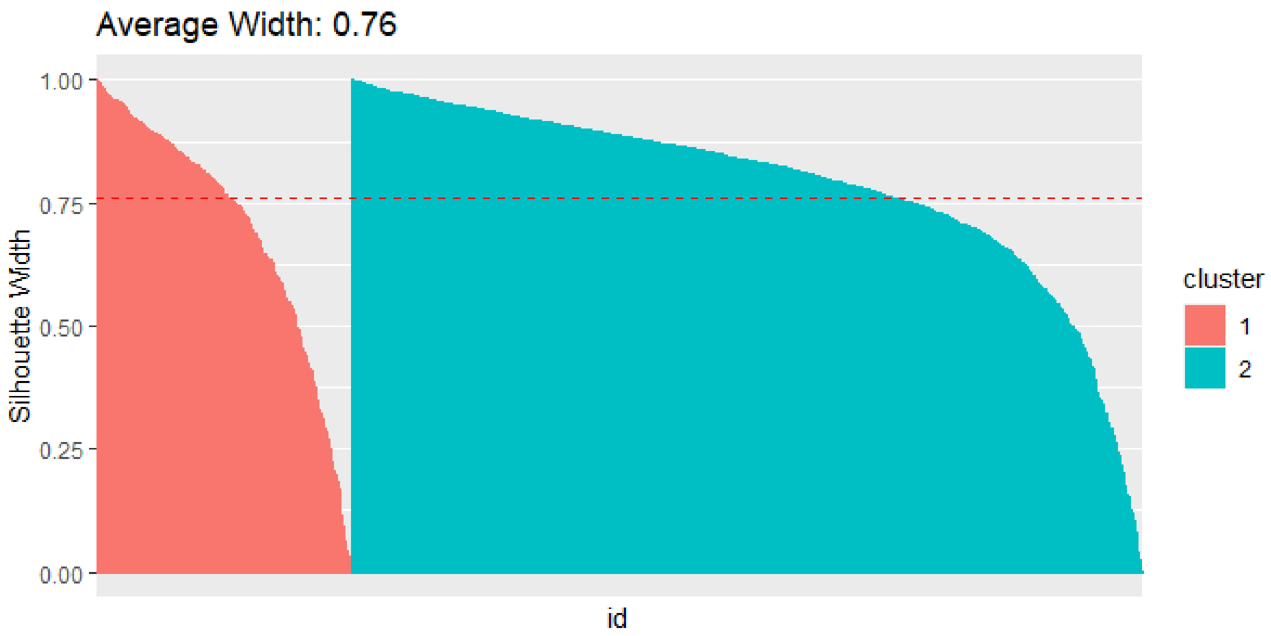

eFigure 6. Average silhouette value of longitudinal clustering of cervical length in twin pregnancies

© 2024 Wu T et al. *JAMA Network Open*

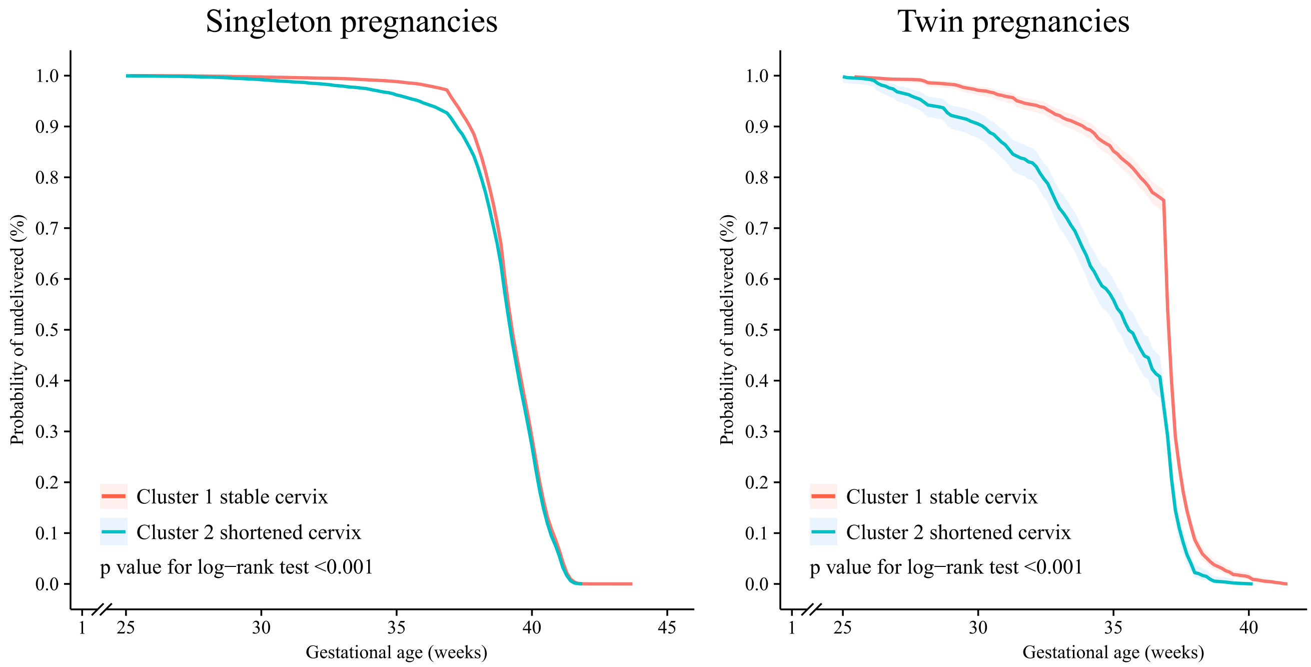

eFigure 7. Survival analysis of gestational age at delivery stratified by cervical length clusters in singleton and twin pregnancies

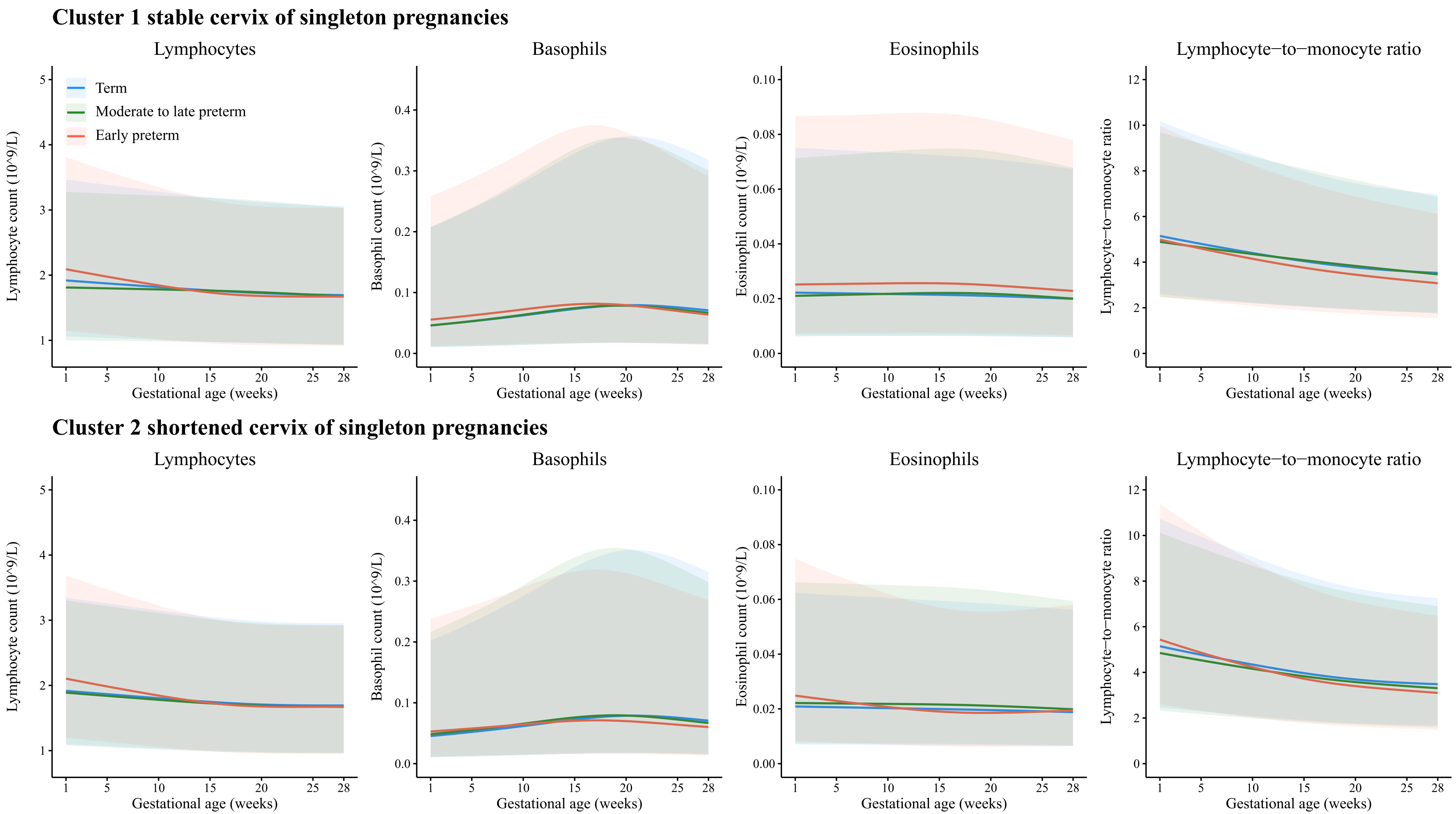

eFigure 8. Gestational age-specific trajectories for lymphocytes, basophils, eosinophils, lymphocyte-to-monocyte ratio stratified by cervical length clusters in singleton pregnancies.

© 2024 Wu T et al. *JAMA Network Open*

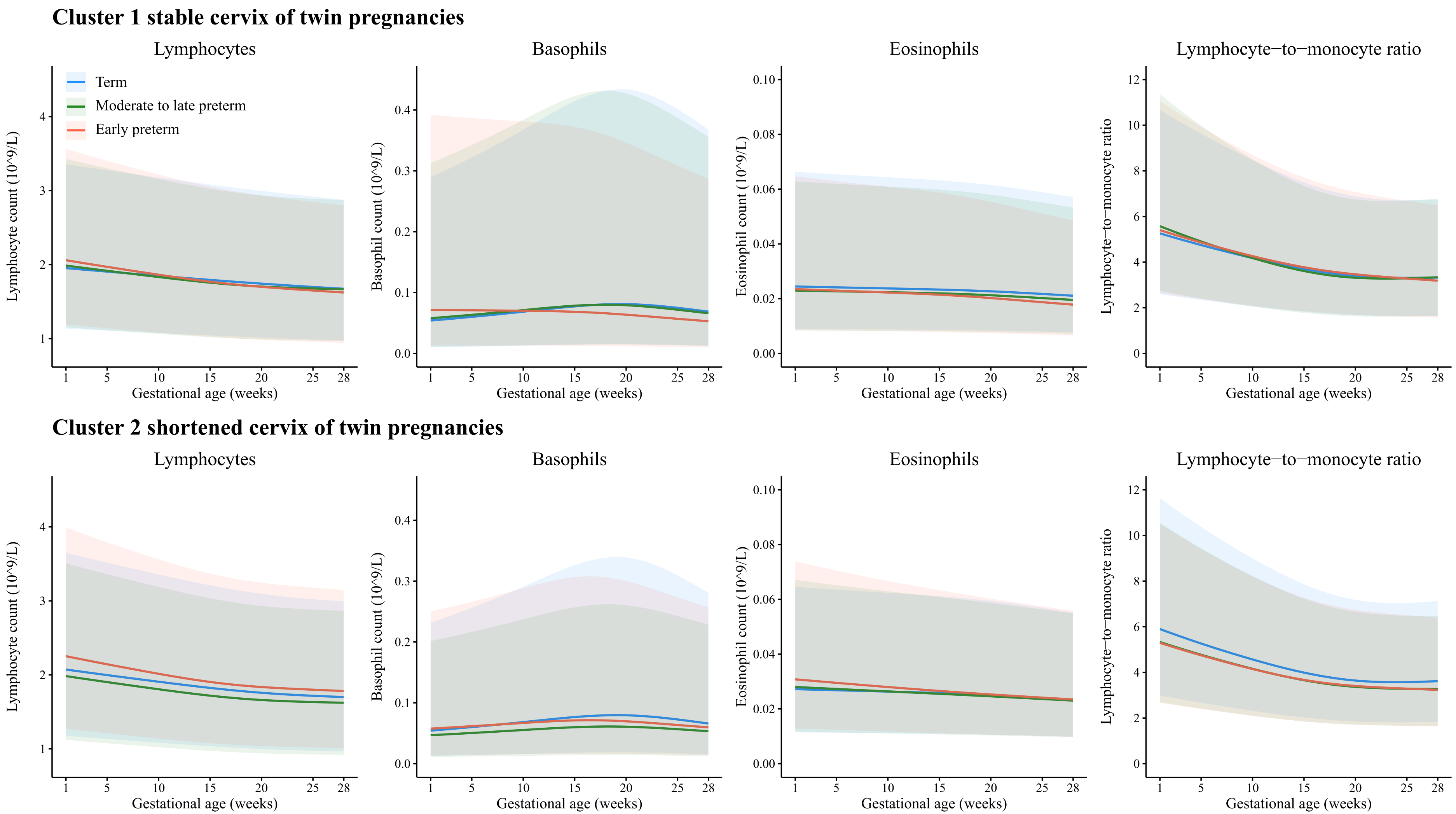

eFigure 9. Gestational age-specific trajectories for lymphocytes, basophils, eosinophils, lymphocyte-to-monocyte ratio stratified by cervical length clusters in twin pregnancies.

© 2024 Wu T et al. *JAMA Network Open*

Cluster 1 stable cervix of singleton pregnancies

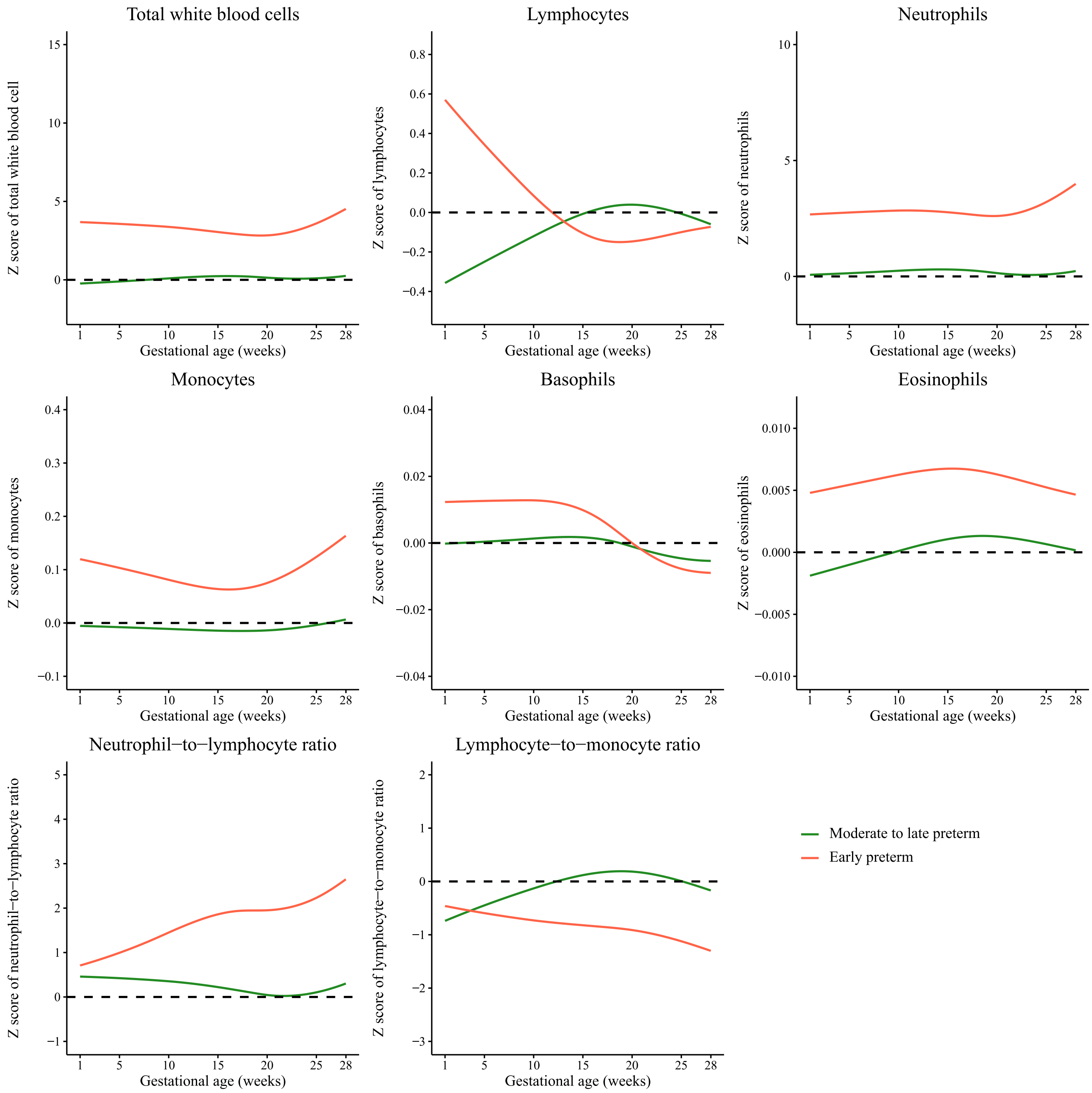

eFigure 10. Gestational age-specific Z-score for white blood cell indicators in singleton pregnancies with stable cervix with reference to term birth

© 2024 Wu T et al. *JAMA Network Open*

Cluster 2 shortened cervix of singleton pregnancies

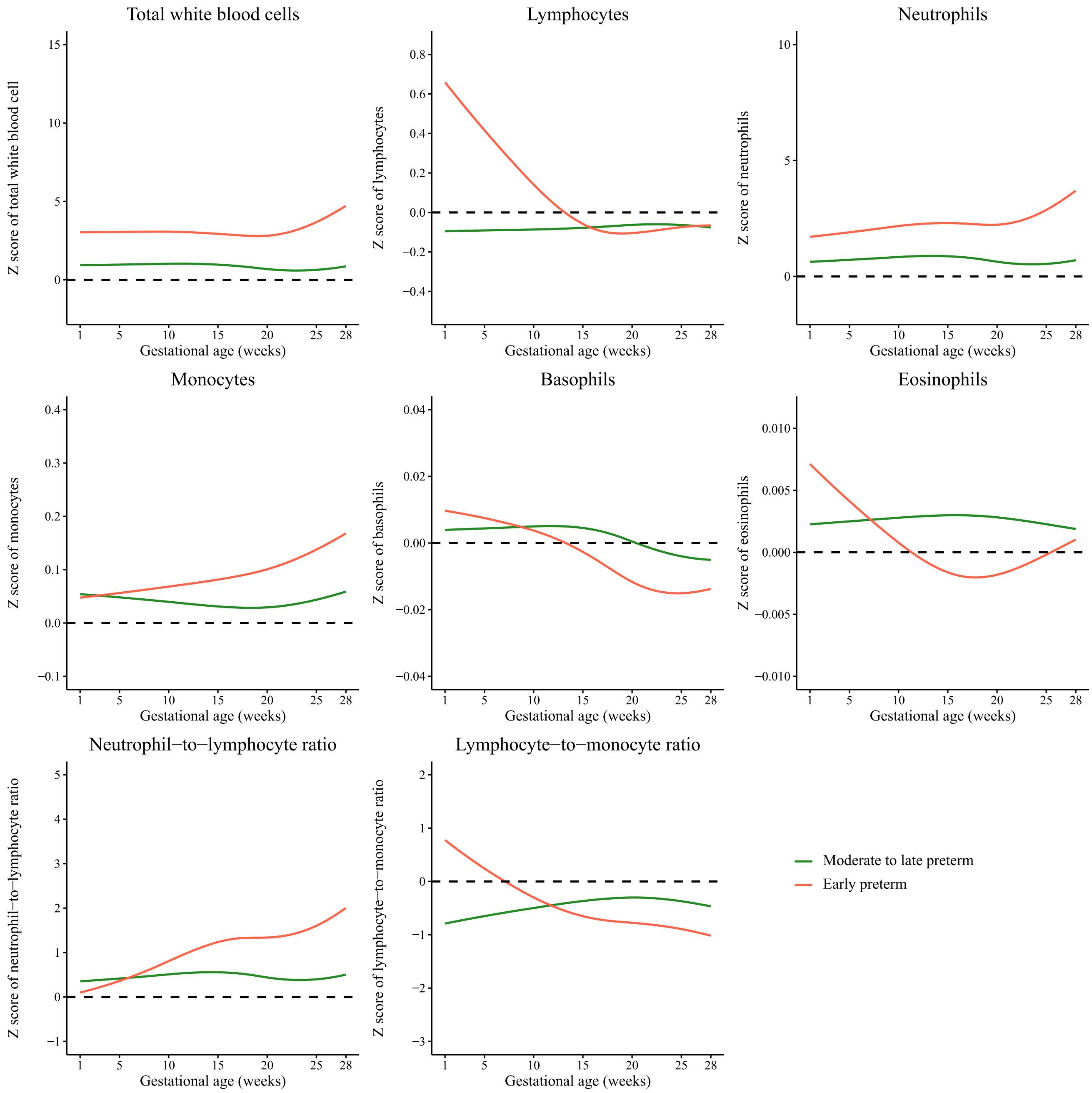

eFigure 11. Gestational age-specific Z-score for white blood cell indicators in singleton pregnancies with shortened cervix with reference to term birth

© 2024 Wu T et al. *JAMA Network Open*

Cluster 1 stable cervix of twin pregnancies

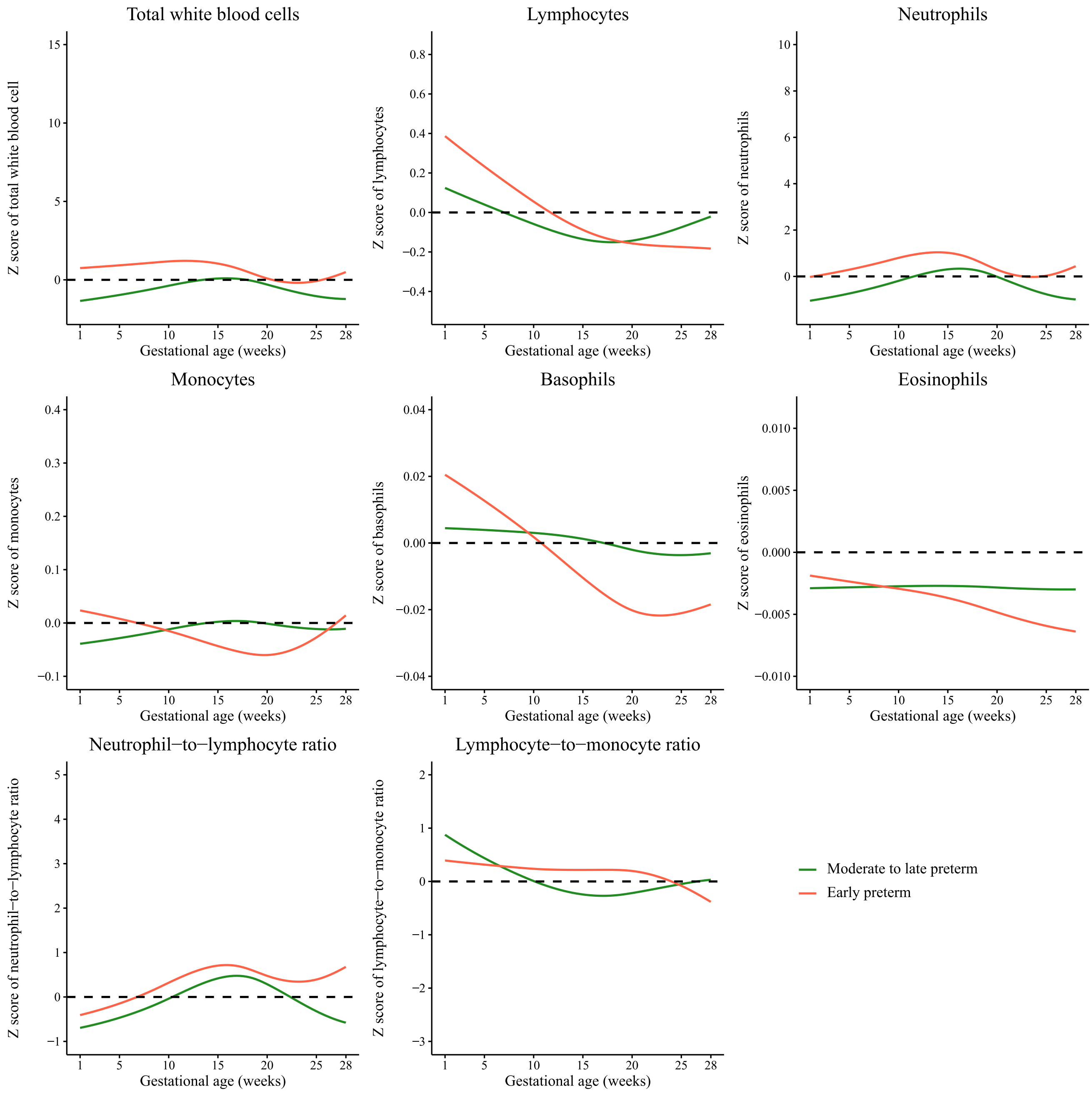

eFigure 12. Gestational age-specific Z-score for white blood cell indicators in twin pregnancies with stable cervix with reference to term birth

© 2024 Wu T et al. *JAMA Network Open*

Cluster 2 shortened cervix of twin pregnancies

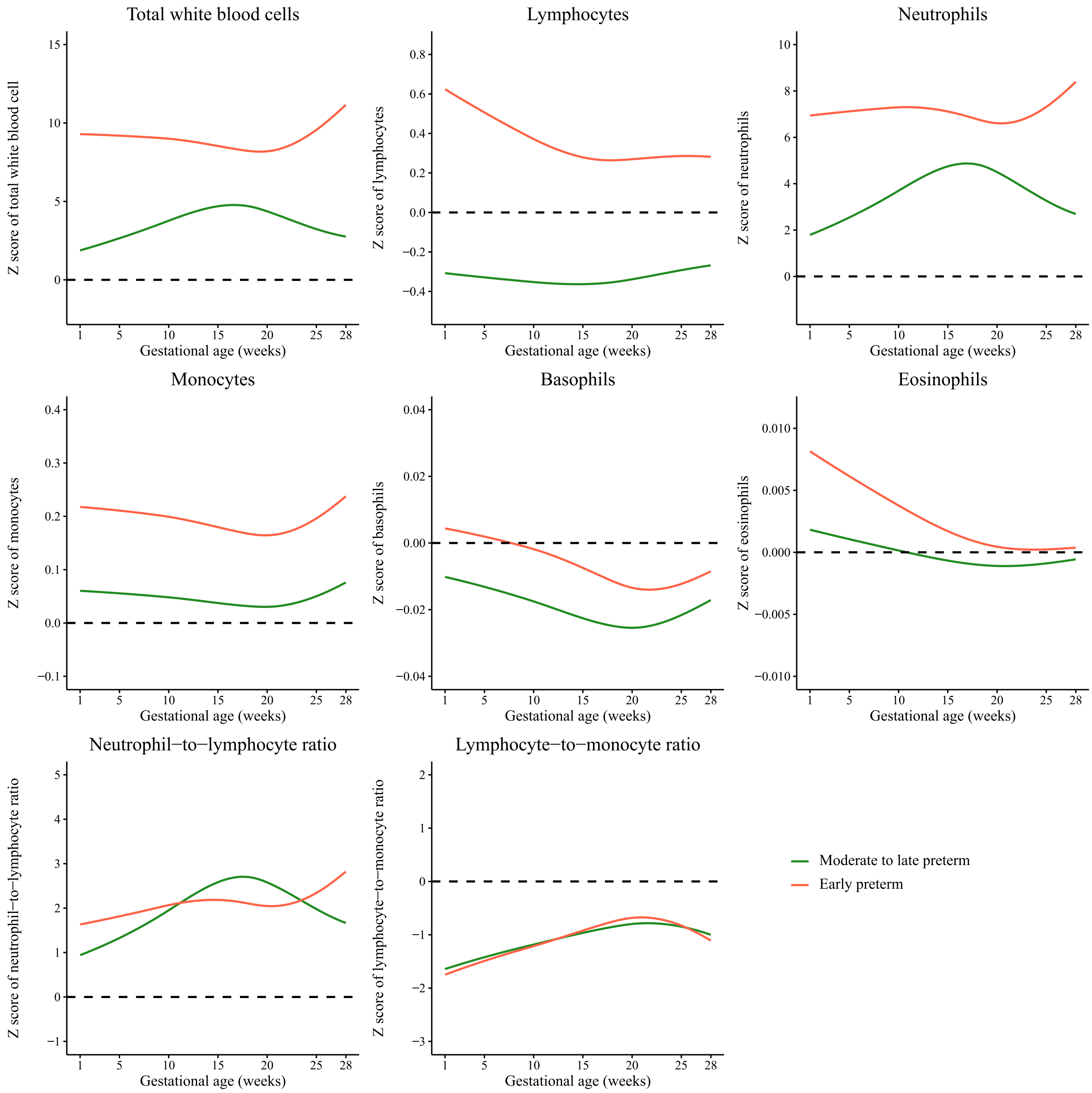

eFigure 13. Gestational age-specific Z-score for white blood cell indicators in twin pregnancies with shortened cervix with reference to term birth

© 2024 Wu T et al. *JAMA Network Open*

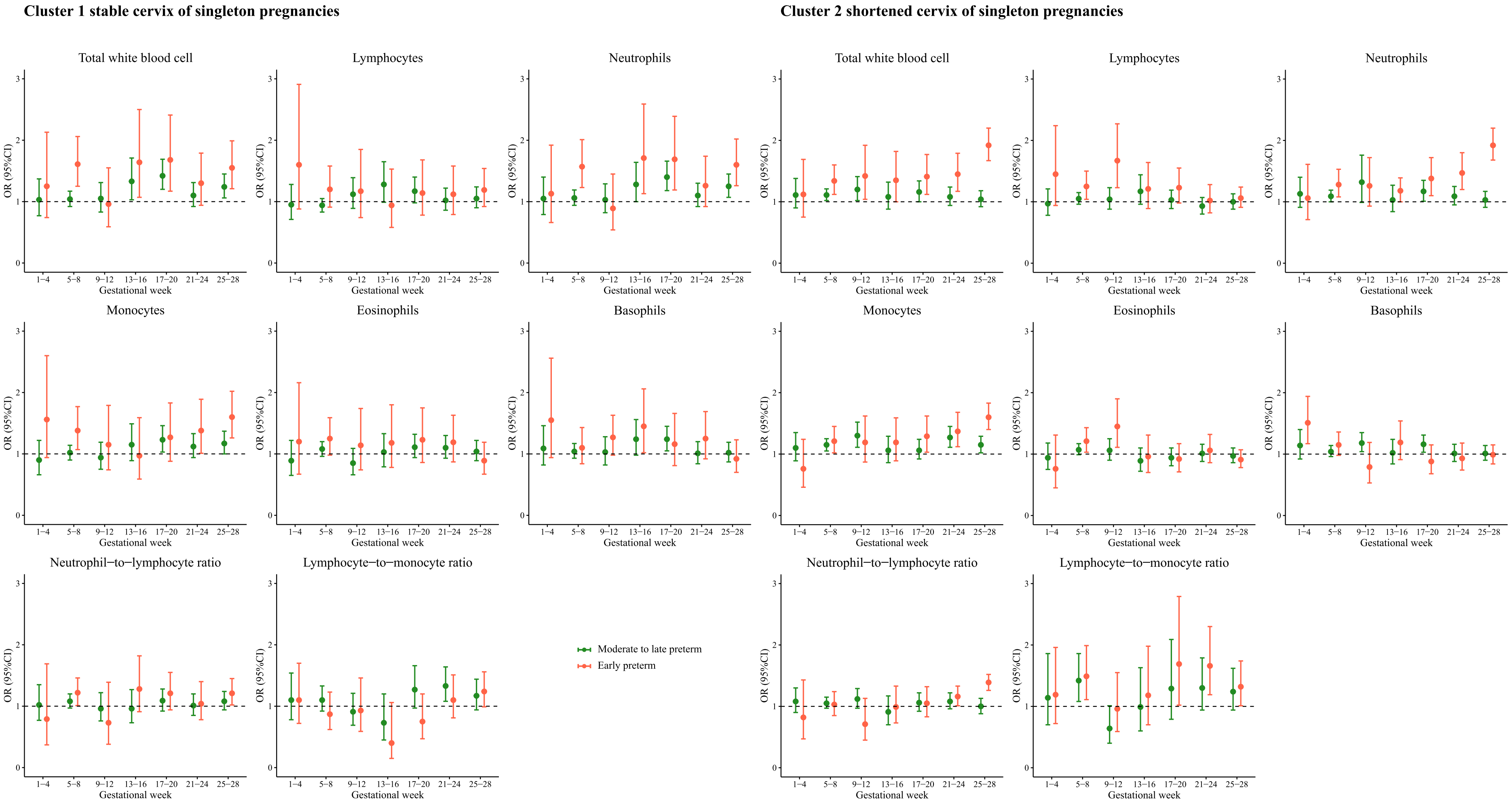

eFigure 14. The relationship between white blood cell indicators and spontaneous preterm birth stratified by cervical length clusters in singleton pregnancies.

© 2024 Wu T et al. *JAMA Network Open*

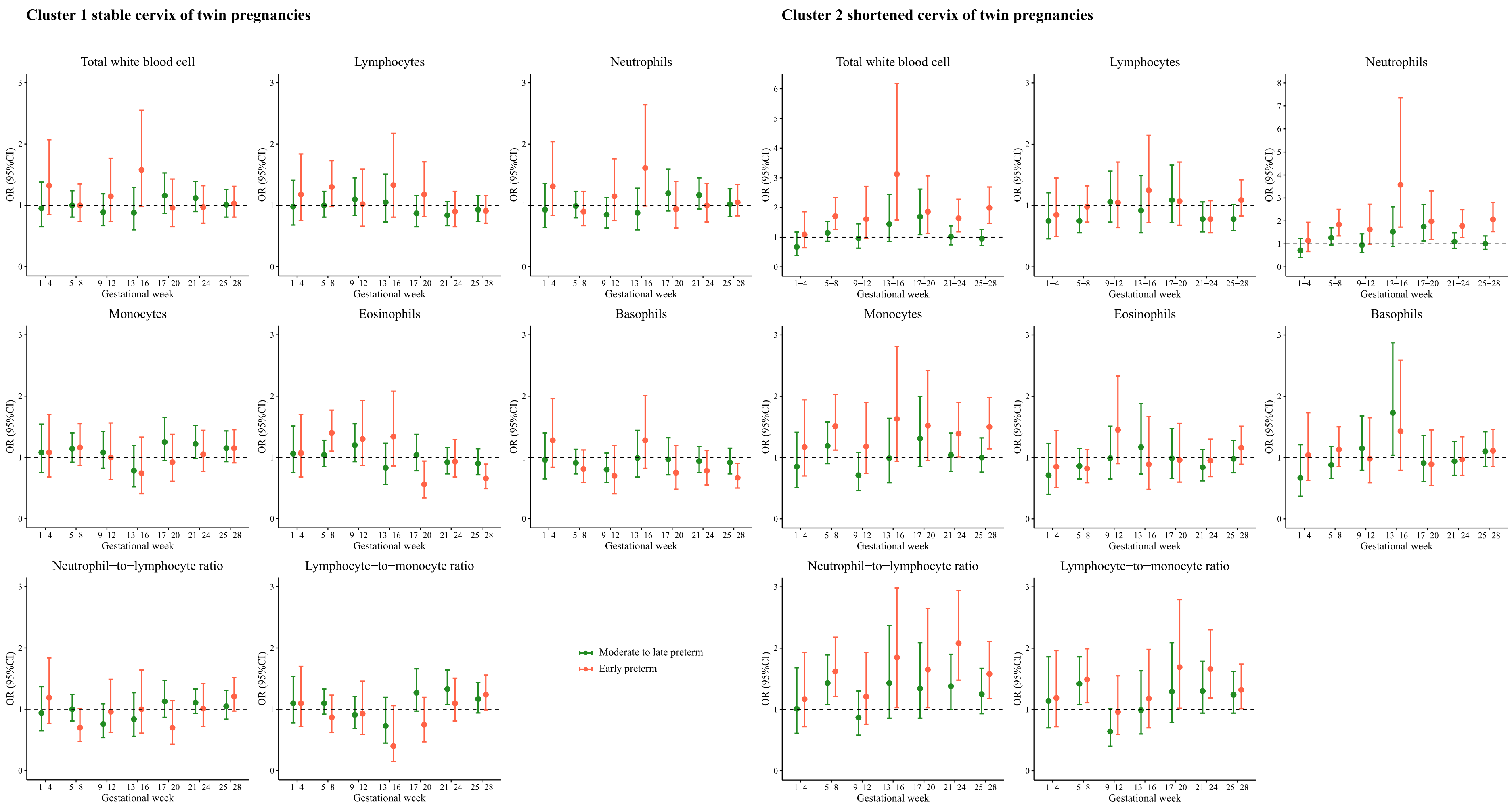

eFigure 15. The relationship between white blood cell indicators and spontaneous preterm birth stratified by cervical length clusters in twin pregnancies.

© 2024 Wu T et al. *JAMA Network Open*

eTable 5. Likelihood ratio test for between-group comparison of white blood cell indicators by gestational week of delivery

White blood cell tests indications	Cervical length trajectory patterns	P value of likelihood ratio test	
		**Singleton pregnancies**	**Twin pregnancies**
Total white blood cell	Cluster 1 stable cervix	**<0.001**	0.204
	Cluster 2 shortened cervix	**<0.001**	**<0.001**
Lymphocytes	Cluster 1 stable cervix	0.285	0.670
	Cluster 2 shortened cervix	0.158	0.017
Neutrophils	Cluster 1 stable cervix	**<0.001**	0.119
	Cluster 2 shortened cervix	**<0.001**	**<0.001**
Monocytes	Cluster 1 stable cervix	0.509	0.502
	Cluster 2 shortened cervix	0.399	0.009
Eosinophils	Cluster 1 stable cervix	0.984	0.360
	Cluster 2 shortened cervix	0.191	0.540
Basophils	Cluster 1 stable cervix	0.208	0.091
	Cluster 2 shortened cervix	0.084	0.616
Neutrophil-to-lymphocyte ratio	Cluster 1 stable cervix	**<0.001**	0.399
	Cluster 2 shortened cervix	**<0.001**	**<0.001**
Lymphocyte-to-monocyte ratio	Cluster 1 stable cervix	**<0.001**	0.073
	Cluster 2 shortened cervix	**<0.001**	**<0.001**

Note: To compare whether there are statistically significant differences in the white blood cell indicators trajectories curves among the early preterm birth, moderate to late preterm birth, and term birth, we fitted white blood cell indicators trajectories curves within each group using a random effects model with natural cubic splines and subsequently employed likelihood ratio tests to assess for statistical differences between the three models. The p-values presented in the above table correspond to the intergroup comparisons derived from the likelihood ratio tests.

© 2024 Wu T et al. *JAMA Network Open*

eTable 6. Estimating the association between white blood cell indicators and spontaneous preterm birth using logistic regression with reference to term birth

White blood cell tests indications	Gestational weeks interval	Spontaneous preterm birth	Cervical length trajectory patterns	Singleton pregnancies			Twin pregnancies		
				OR (95%CI)	P for OR	P for interaction	OR (95%CI)	P for OR	P for interaction
Total white blood cells	1~4	Moderate to late preterm	Cluster 1 stable cervix	1.03 (0.77~1.37)	0.85	0.38	0.95 (0.65~1.38)	0.77	0.17
			Cluster 2 shortened cervix	1.11 (0.90~1.38)	0.34		0.67 (0.39~1.17)	0.16	
		Early preterm	Cluster 1 stable cervix	1.25 (0.74~2.13)	0.40	0.16	1.32 (0.85~2.07)	0.22	0.28
			Cluster 2 shortened cervix	1.12 (0.75~1.69)	0.57		1.09 (0.64~1.86)	0.74	
	5~8	Moderate to late preterm	Cluster 1 stable cervix	1.04 (0.92~1.17)	0.54	<0.01	1.00 (0.81~1.24)	0.99	0.55
			Cluster 2 shortened cervix	**1.11 (1.02~1.21)**	**0.02**		1.15 (0.86~1.53)	0.36	
		Early preterm	Cluster 1 stable cervix	**1.61 (1.25~2.06)**	**<0.01**	<0.01	1.00 (0.74~1.35)	0.99	<0.01
			Cluster 2 shortened cervix	**1.34 (1.12~1.60)**	**<0.01**		**1.71 (1.26~2.34)**	**<0.01**	
	9~12	Moderate to late preterm	Cluster 1 stable cervix	1.05 (0.83~1.31)	0.70	<0.01	0.89 (0.67~1.19)	0.44	0.85
			Cluster 2 shortened cervix	**1.20 (1.02~1.41)**	**0.03**		0.96 (0.63~1.45)	0.85	
		Early preterm	Cluster 1 stable cervix	0.96 (0.59~1.55)	0.85	0.02	1.15 (0.74~1.77)	0.53	0.33
			Cluster 2 shortened cervix	**1.42 (1.04~1.92)**	**0.03**		1.61 (0.96~2.71)	0.07	
	13~16	Moderate to late preterm	Cluster 1 stable cervix	**1.33 (1.03~1.71)**	**0.03**	0.11	0.88 (0.60~1.29)	0.50	0.71
			Cluster 2 shortened cervix	1.08 (0.88~1.32)	0.48		1.44 (0.85~2.45)	0.18	
		Early preterm	Cluster 1 stable cervix	**1.64 (1.07~2.50)**	**0.02**	<0.01	1.58 (0.98~2.55)	0.06	<0.01
			Cluster 2 shortened cervix	**1.35 (1.00~1.82)**	**0.05**		**3.13 (1.58~6.18)**	**<0.01**	
	17~20	Moderate to late preterm	Cluster 1 stable cervix	**1.42 (1.20~1.69)**	**<0.01**	<0.01	1.16 (0.87~1.53)	0.31	<0.01
			Cluster 2 shortened cervix	**1.16 (1.00~1.34)**	**0.05**		**1.69 (1.09~2.62)**	**0.02**	
		Early preterm	Cluster 1 stable cervix	**1.68 (1.17~2.41)**	**<0.01**	<0.01	0.96 (0.65~1.43)	0.85	0.03
			Cluster 2 shortened cervix	**1.41 (1.12~1.77)**	**<0.01**		**1.86 (1.13~3.07)**	**0.02**	
	21~24	Moderate to late preterm	Cluster 1 stable cervix	1.10 (0.92~1.31)	0.28	0.20	1.12 (0.90~1.39)	0.31	0.42
			Cluster 2 shortened cervix	1.08 (0.94~1.24)	0.31		1.02 (0.74~1.38)	0.92	
		Early preterm	Cluster 1 stable cervix	1.30 (0.94~1.79)	0.11	<0.01	0.97 (0.71~1.32)	0.83	<0.01
			Cluster 2 shortened cervix	**1.45 (1.17~1.79)**	**<0.01**		**1.64 (1.18~2.28)**	**<0.01**	
	25~28	Moderate to late preterm	Cluster 1 stable cervix	**1.24 (1.06~1.45)**	**<0.01**	0.22	1.01 (0.81~1.26)	0.93	0.52
			Cluster 2 shortened cervix	1.04 (0.92~1.18)	0.54		0.95 (0.72~1.26)	0.74	
		Early preterm	Cluster 1 stable cervix	**1.55 (1.21~1.99)**	**<0.01**	<0.01	1.03 (0.81~1.31)	0.79	<0.01
			Cluster 2 shortened cervix	**1.92 (1.67~2.20)**	**<0.01**		**1.99 (1.47~2.69)**	**<0.01**	
Lymphocytes	1~4	Moderate to late preterm	Cluster 1 stable cervix	0.95 (0.71~1.28)	0.75	0.70	0.98 (0.68~1.41)	0.92	0.29
			Cluster 2 shortened cervix	0.97 (0.78~1.21)	0.81		0.75 (0.46~1.21)	0.23	
		Early preterm	Cluster 1 stable cervix	1.60 (0.88~2.91)	0.13	0.81	1.18 (0.75~1.84)	0.47	0.86
			Cluster 2 shortened cervix	1.45 (0.94~2.24)	0.09		0.85 (0.50~1.45)	0.55	
	5~8	Moderate to late preterm	Cluster 1 stable cervix	0.94 (0.83~1.05)	0.27	0.40	1.00 (0.81~1.23)	0.97	0.08
			Cluster 2 shortened cervix	1.05 (0.96~1.15)	0.25		0.75 (0.56~1.00)	0.05	
		Early preterm	Cluster 1 stable cervix	1.20 (0.91~1.58)	0.19	<0.01	1.30 (0.98~1.73)	0.07	0.37

© 2024 Wu T et al. *JAMA Network Open*

			Cluster 2 shortened cervix	**1.25 (1.04~1.50)**	**0.02**		0.98 (0.73~1.32)	0.89	
	9~12	Moderate to late preterm	Cluster 1 stable cervix	1.12 (0.89~1.39)	0.34	0.30	1.10 (0.84~1.45)	0.48	0.44
			Cluster 2 shortened cervix	1.04 (0.88~1.23)	0.63		1.06 (0.73~1.56)	0.75	
		Early preterm	Cluster 1 stable cervix	1.17 (0.74~1.85)	0.51	<0.01	1.02 (0.66~1.59)	0.92	0.72
			Cluster 2 shortened cervix	**1.67 (1.23~2.27)**	**<0.01**		1.05 (0.64~1.71)	0.86	
	13~16	Moderate to late preterm	Cluster 1 stable cervix	1.28 (0.99~1.65)	0.06	0.71	1.05 (0.73~1.51)	0.81	0.75
			Cluster 2 shortened cervix	1.17 (0.96~1.44)	0.12		0.92 (0.56~1.49)	0.73	
		Early preterm	Cluster 1 stable cervix	0.94 (0.58~1.53)	0.81	0.29	1.33 (0.81~2.18)	0.25	0.28
			Cluster 2 shortened cervix	1.21 (0.89~1.64)	0.23		1.25 (0.72~2.15)	0.43	
	17~20	Moderate to late preterm	Cluster 1 stable cervix	1.17 (0.98~1.40)	0.09	0.13	0.87 (0.65~1.16)	0.35	0.85
			Cluster 2 shortened cervix	1.03 (0.89~1.19)	0.70		1.09 (0.72~1.66)	0.67	
		Early preterm	Cluster 1 stable cervix	1.14 (0.78~1.68)	0.50	0.66	1.18 (0.82~1.71)	0.38	0.60
			Cluster 2 shortened cervix	1.23 (0.98~1.55)	0.07		1.07 (0.68~1.71)	0.76	
	21~24	Moderate to late preterm	Cluster 1 stable cervix	1.02 (0.86~1.22)	0.79	0.30	0.84 (0.67~1.06)	0.15	0.11
			Cluster 2 shortened cervix	0.93 (0.80~1.07)	0.32		0.78 (0.57~1.06)	0.11	
		Early preterm	Cluster 1 stable cervix	1.12 (0.79~1.58)	0.52	0.87	0.90 (0.65~1.23)	0.50	0.08
			Cluster 2 shortened cervix	1.02 (0.82~1.28)	0.86		0.78 (0.56~1.08)	0.13	
	25~28	Moderate to late preterm	Cluster 1 stable cervix	1.05 (0.90~1.24)	0.51	0.92	0.93 (0.74~1.16)	0.52	0.05
			Cluster 2 shortened cervix	1.00 (0.88~1.13)	0.95		0.78 (0.59~1.02)	0.07	
		Early preterm	Cluster 1 stable cervix	1.19 (0.92~1.54)	0.20	0.34	0.91 (0.71~1.16)	0.43	0.57
			Cluster 2 shortened cervix	1.06 (0.91~1.24)	0.43		1.09 (0.83~1.42)	0.53	
Neutrophils	1~4	Moderate to late preterm	Cluster 1 stable cervix	1.05 (0.79~1.40)	0.73	0.27	0.93 (0.64~1.36)	0.72	0.21
			Cluster 2 shortened cervix	1.13 (0.91~1.40)	0.25		0.72 (0.41~1.24)	0.23	
		Early preterm	Cluster 1 stable cervix	1.13 (0.66~1.92)	0.66	0.32	1.31 (0.84~2.04)	0.24	0.24
			Cluster 2 shortened cervix	1.06 (0.71~1.61)	0.77		1.14 (0.67~1.94)	0.62	
	5~8	Moderate to late preterm	Cluster 1 stable cervix	1.06 (0.94~1.19)	0.34	<0.01	0.99 (0.80~1.23)	0.94	0.28
			Cluster 2 shortened cervix	**1.09 (1.00~1.19)**	**0.05**		1.27 (0.95~1.70)	0.11	
		Early preterm	Cluster 1 stable cervix	**1.57 (1.23~2.01)**	**<0.01**	<0.01	0.90 (0.67~1.23)	0.52	<0.01
			Cluster 2 shortened cervix	**1.28 (1.08~1.53)**	**<0.01**		**1.84 (1.35~2.50)**	**<0.01**	
	9~12	Moderate to late preterm	Cluster 1 stable cervix	1.03 (0.82~1.29)	0.80	0.26	0.85 (0.63~1.13)	0.26	0.98
			Cluster 2 shortened cervix	**1.18 (1.00~1.39)**	**0.04**		0.95 (0.63~1.44)	0.83	
		Early preterm	Cluster 1 stable cervix	0.89 (0.54~1.45)	0.63	0.16	1.15 (0.75~1.76)	0.53	0.30
			Cluster 2 shortened cervix	1.26 (0.93~1.72)	0.14		1.63 (0.98~2.73)	0.06	
	13~16	Moderate to late preterm	Cluster 1 stable cervix	**1.28 (1.00~1.64)**	**0.05**	0.27	0.88 (0.60~1.28)	0.50	0.67
			Cluster 2 shortened cervix	1.03 (0.84~1.27)	0.75		1.53 (0.89~2.61)	0.12	
		Early preterm	Cluster 1 stable cervix	**1.71 (1.13~2.59)**	**0.01**	<0.01	1.61 (0.99~2.64)	0.06	<0.01
			Cluster 2 shortened cervix	1.32 (0.99~1.76)	0.06		**3.57 (1.73~7.36)**	**<0.01**	
	17~20	Moderate to late preterm	Cluster 1 stable cervix	**1.40 (1.18~1.66)**	**<0.01**	<0.01	1.20 (0.91~1.59)	0.20	<0.01
			Cluster 2 shortened cervix	**1.17 (1.01~1.35)**	**0.04**		**1.75 (1.13~2.72)**	**0.01**	
		Early preterm	Cluster 1 stable cervix	**1.69 (1.19~2.39)**	**<0.01**	<0.01	0.94 (0.63~1.39)	0.75	0.02

© 2024 Wu T et al. *JAMA Network Open*

			Cluster 2 shortened cervix	**1.38 (1.10~1.72)**	**<0.01**		**1.98 (1.19~3.31)**	**<0.01**	
	21~24	Moderate to late preterm	Cluster 1 stable cervix	1.10 (0.92~1.30)	0.30	0.15	1.17 (0.94~1.45)	0.16	0.19
			Cluster 2 shortened cervix	1.09 (0.95~1.25)	0.24		1.10 (0.81~1.49)	0.56	
		Early preterm	Cluster 1 stable cervix	1.26 (0.92~1.74)	0.15	<0.01	1.00 (0.73~1.36)	0.98	<0.01
			Cluster 2 shortened cervix	**1.47 (1.20~1.80)**	**<0.01**		**1.78 (1.27~2.48)**	**<0.01**	
	25~28	Moderate to late preterm	Cluster 1 stable cervix	**1.25 (1.07~1.45)**	**<0.01**	0.21	1.02 (0.82~1.27)	0.85	0.78
			Cluster 2 shortened cervix	1.03 (0.91~1.17)	0.59		1.01 (0.76~1.35)	0.92	
		Early preterm	Cluster 1 stable cervix	**1.60 (1.26~2.02)**	**<0.01**	<0.01	1.05 (0.83~1.34)	0.67	<0.01
			Cluster 2 shortened cervix	**1.92 (1.68~2.20)**	**<0.01**		**2.07 (1.53~2.81)**	**<0.01**	
Monocytes	1~4	Moderate to late preterm	Cluster 1 stable cervix	0.90 (0.66~1.22)	0.48	0.56	1.08 (0.75~1.54)	0.68	0.84
			Cluster 2 shortened cervix	1.10 (0.89~1.35)	0.38		0.85 (0.51~1.41)	0.53	
		Early preterm	Cluster 1 stable cervix	1.56 (0.94~2.60)	0.08	0.62	1.08 (0.68~1.70)	0.76	0.36
			Cluster 2 shortened cervix	0.76 (0.46~1.24)	0.27		1.17 (0.70~1.94)	0.55	
	5~8	Moderate to late preterm	Cluster 1 stable cervix	1.02 (0.90~1.14)	0.79	<0.01	1.14 (0.92~1.40)	0.23	0.10
			Cluster 2 shortened cervix	**1.15 (1.05~1.25)**	**<0.01**		1.19 (0.90~1.58)	0.22	
		Early preterm	Cluster 1 stable cervix	**1.38 (1.07~1.77)**	**0.01**	<0.01	1.16 (0.87~1.55)	0.31	<0.01
			Cluster 2 shortened cervix	**1.21 (1.02~1.45)**	**0.03**		**1.51 (1.12~2.03)**	**<0.01**	
	9~12	Moderate to late preterm	Cluster 1 stable cervix	0.94 (0.75~1.19)	0.63	<0.01	1.08 (0.82~1.42)	0.60	0.42
			Cluster 2 shortened cervix	**1.30 (1.11~1.52)**	**<0.01**		0.71 (0.46~1.08)	0.11	
		Early preterm	Cluster 1 stable cervix	1.15 (0.74~1.79)	0.53	0.20	1.00 (0.64~1.56)	0.99	0.23
			Cluster 2 shortened cervix	1.19 (0.87~1.62)	0.29		1.18 (0.74~1.90)	0.49	
	13~16	Moderate to late preterm	Cluster 1 stable cervix	1.15 (0.89~1.49)	0.27	0.42	0.78 (0.52~1.19)	0.25	0.57
			Cluster 2 shortened cervix	1.06 (0.86~1.29)	0.60		0.99 (0.59~1.64)	0.96	
		Early preterm	Cluster 1 stable cervix	0.97 (0.59~1.59)	0.89	0.29	0.74 (0.41~1.33)	0.31	0.65
			Cluster 2 shortened cervix	1.19 (0.89~1.59)	0.23		1.63 (0.94~2.81)	0.08	
	17~20	Moderate to late preterm	Cluster 1 stable cervix	**1.23 (1.03~1.46)**	**0.02**	0.16	1.25 (0.95~1.65)	0.12	0.83
			Cluster 2 shortened cervix	1.06 (0.92~1.24)	0.42		1.31 (0.85~2.00)	0.22	
		Early preterm	Cluster 1 stable cervix	1.27 (0.88~1.83)	0.20	0.01	0.92 (0.61~1.38)	0.68	0.13
			Cluster 2 shortened cervix	**1.29 (1.03~1.62)**	**0.03**		1.52 (0.95~2.42)	0.08	
	21~24	Moderate to late preterm	Cluster 1 stable cervix	1.12 (0.94~1.33)	0.21	<0.01	1.22 (0.98~1.52)	0.07	0.23
			Cluster 2 shortened cervix	**1.27 (1.11~1.45)**	**<0.01**		1.04 (0.77~1.40)	0.81	
		Early preterm	Cluster 1 stable cervix	**1.38 (1.01~1.89)**	**0.04**	<0.01	1.05 (0.77~1.44)	0.75	<0.01
			Cluster 2 shortened cervix	**1.37 (1.12~1.68)**	**<0.01**		1.39 (1.01~1.90)	0.04	
	25~28	Moderate to late preterm	Cluster 1 stable cervix	**1.17 (1.00~1.37)**	**0.05**	0.02	1.15 (0.93~1.43)	0.21	0.62
			Cluster 2 shortened cervix	**1.15 (1.02~1.29)**	**0.03**		1.00 (0.76~1.32)	0.99	
		Early preterm	Cluster 1 stable cervix	**1.60 (1.26~2.02)**	**<0.01**	<0.01	1.15 (0.91~1.45)	0.26	<0.01
			Cluster 2 shortened cervix	**1.60 (1.40~1.83)**	**<0.01**		**1.50 (1.14~1.98)**	**<0.01**	
Eosinophils	1~4	Moderate to late preterm	Cluster 1 stable cervix	0.89 (0.65~1.22)	0.46	0.43	1.06 (0.75~1.51)	0.73	0.48
			Cluster 2 shortened cervix	0.94 (0.75~1.18)	0.62		0.71 (0.40~1.23)	0.22	
		Early preterm	Cluster 1 stable cervix	1.20 (0.67~2.16)	0.54	0.53	1.07 (0.68~1.70)	0.77	0.93

© 2024 Wu T et al. *JAMA Network Open*

			Cluster 2 shortened cervix	0.76 (0.45~1.31)	0.33		0.85 (0.51~1.44)	0.55	
	5~8	Moderate to late preterm	Cluster 1 stable cervix	1.08 (0.96~1.20)	0.20	0.89	1.04 (0.85~1.28)	0.71	0.61
			Cluster 2 shortened cervix	1.07 (0.99~1.17)	0.10		0.86 (0.65~1.15)	0.31	
		Early preterm	Cluster 1 stable cervix	1.25 (0.98~1.59)	0.08	<0.01	**1.40 (1.10~1.77)**	**<0.01**	0.95
			Cluster 2 shortened cervix	**1.21 (1.03~1.43)**	**0.02**		0.82 (0.59~1.13)	0.22	
	9~12	Moderate to late preterm	Cluster 1 stable cervix	0.85 (0.66~1.09)	0.20	0.71	1.20 (0.93~1.55)	0.17	0.29
			Cluster 2 shortened cervix	1.06 (0.90~1.25)	0.48		0.99 (0.65~1.51)	0.98	
		Early preterm	Cluster 1 stable cervix	1.14 (0.74~1.74)	0.55	<0.01	1.30 (0.87~1.93)	0.20	0.59
			Cluster 2 shortened cervix	**1.45 (1.11~1.90)**	**<0.01**		1.45 (0.90~2.33)	0.12	
	13~16	Moderate to late preterm	Cluster 1 stable cervix	1.03 (0.79~1.33)	0.83	0.49	0.83 (0.56~1.23)	0.35	0.97
			Cluster 2 shortened cervix	0.89 (0.72~1.10)	0.29		1.17 (0.73~1.88)	0.51	
		Early preterm	Cluster 1 stable cervix	1.18 (0.78~1.80)	0.43	0.88	1.34 (0.86~2.08)	0.19	0.63
			Cluster 2 shortened cervix	0.96 (0.70~1.31)	0.80		0.89 (0.48~1.67)	0.73	
	17~20	Moderate to late preterm	Cluster 1 stable cervix	1.11 (0.94~1.32)	0.22	0.96	1.04 (0.78~1.38)	0.79	0.99
			Cluster 2 shortened cervix	0.94 (0.81~1.10)	0.45		0.99 (0.66~1.47)	0.95	
		Early preterm	Cluster 1 stable cervix	1.23 (0.86~1.75)	0.26	0.77	**0.56 (0.34~0.94)**	**0.03**	0.14
			Cluster 2 shortened cervix	0.92 (0.71~1.17)	0.49		0.96 (0.60~1.56)	0.88	
	21~24	Moderate to late preterm	Cluster 1 stable cervix	1.10 (0.93~1.30)	0.28	0.50	0.92 (0.73~1.16)	0.47	0.19
			Cluster 2 shortened cervix	1.01 (0.88~1.16)	0.89		0.84 (0.62~1.13)	0.24	
		Early preterm	Cluster 1 stable cervix	1.19 (0.87~1.63)	0.28	0.29	0.93 (0.68~1.29)	0.68	0.57
			Cluster 2 shortened cervix	1.06 (0.86~1.32)	0.57		0.95 (0.69~1.30)	0.74	
	25~28	Moderate to late preterm	Cluster 1 stable cervix	1.04 (0.89~1.22)	0.61	0.73	0.90 (0.72~1.14)	0.39	0.44
			Cluster 2 shortened cervix	0.97 (0.86~1.10)	0.62		0.98 (0.75~1.28)	0.88	
		Early preterm	Cluster 1 stable cervix	0.89 (0.67~1.19)	0.43	0.11	**0.66 (0.49~0.89)**	**<0.01**	0.55
			Cluster 2 shortened cervix	0.91 (0.78~1.07)	0.25		1.16 (0.89~1.51)	0.28	
Basophils	1~4	Moderate to late preterm	Cluster 1 stable cervix	1.09 (0.82~1.46)	0.55	0.24	0.96 (0.65~1.40)	0.82	0.19
			Cluster 2 shortened cervix	1.14 (0.92~1.40)	0.23		0.67 (0.37~1.21)	0.18	
		Early preterm	Cluster 1 stable cervix	1.55 (0.94~2.56)	0.09	<0.01	1.28 (0.84~1.96)	0.24	0.42
			Cluster 2 shortened cervix	**1.51 (1.17~1.94)**	**<0.01**		1.04 (0.63~1.73)	0.87	
	5~8	Moderate to late preterm	Cluster 1 stable cervix	1.04 (0.93~1.17)	0.47	0.06	0.91 (0.73~1.13)	0.40	0.25
			Cluster 2 shortened cervix	1.04 (0.96~1.14)	0.33		0.88 (0.66~1.18)	0.40	
		Early preterm	Cluster 1 stable cervix	1.10 (0.84~1.43)	0.50	0.82	0.81 (0.59~1.12)	0.21	0.97
			Cluster 2 shortened cervix	1.15 (0.98~1.36)	0.09		1.13 (0.85~1.50)	0.39	
	9~12	Moderate to late preterm	Cluster 1 stable cervix	1.03 (0.82~1.28)	0.80	<0.01	0.80 (0.59~1.07)	0.13	0.98
			Cluster 2 shortened cervix	**1.18 (1.04~1.35)**	**0.01**		1.15 (0.79~1.68)	0.47	
		Early preterm	Cluster 1 stable cervix	1.27 (0.98~1.63)	0.07	0.71	0.70 (0.41~1.19)	0.18	0.33
			Cluster 2 shortened cervix	0.79 (0.53~1.19)	0.26		0.98 (0.59~1.65)	0.95	
	13~16	Moderate to late preterm	Cluster 1 stable cervix	1.24 (0.98~1.56)	0.07	0.12	0.99 (0.68~1.44)	0.95	0.08
			Cluster 2 shortened cervix	1.02 (0.84~1.24)	0.85		**1.73 (1.04~2.87)**	**0.04**	
		Early preterm	Cluster 1 stable cervix	**1.45 (1.02~2.06)**	**0.04**	0.03	1.28 (0.82~2.01)	0.28	0.16

© 2024 Wu T et al. *JAMA Network Open*

			Cluster 2 shortened cervix	1.19 (0.91~1.54)	0.20		1.43 (0.79~2.59)	0.24	
	17~20	Moderate to late preterm	Cluster 1 stable cervix	**1.24 (1.05~1.45)**	**<0.01**	<0.01	0.97 (0.72~1.32)	0.85	0.35
			Cluster 2 shortened cervix	**1.16 (1.03~1.31)**	**0.01**		0.91 (0.61~1.36)	0.64	
		Early preterm	Cluster 1 stable cervix	1.16 (0.81~1.66)	0.41	0.63	0.75 (0.48~1.19)	0.22	0.35
			Cluster 2 shortened cervix	0.88 (0.68~1.15)	0.36		0.89 (0.54~1.45)	0.64	
	21~24	Moderate to late preterm	Cluster 1 stable cervix	1.01 (0.84~1.20)	0.95	0.82	0.94 (0.75~1.18)	0.59	0.64
			Cluster 2 shortened cervix	1.01 (0.88~1.16)	0.88		0.94 (0.71~1.26)	0.70	
		Early preterm	Cluster 1 stable cervix	1.25 (0.92~1.69)	0.15	0.84	0.78 (0.55~1.11)	0.17	0.40
			Cluster 2 shortened cervix	0.93 (0.74~1.18)	0.57		0.97 (0.71~1.34)	0.87	
	25~28	Moderate to late preterm	Cluster 1 stable cervix	1.02 (0.87~1.19)	0.84	0.80	0.92 (0.73~1.15)	0.46	0.87
			Cluster 2 shortened cervix	1.01 (0.90~1.14)	0.83		1.10 (0.85~1.42)	0.48	
		Early preterm	Cluster 1 stable cervix	0.92 (0.70~1.23)	0.59	0.71	**0.67 (0.50~0.90)**	**<0.01**	0.46
			Cluster 2 shortened cervix	0.99 (0.84~1.15)	0.85		1.11 (0.85~1.46)	0.43	
Neutrophil-to-lymphocyte ratio	1~4	Moderate to late preterm	Cluster 1 stable subgroup	1.02 (0.77~1.35)	0.89	0.75	0.94 (0.65~1.37)	0.75	0.93
			Cluster 2 shortened subgroup	1.08 (0.90~1.30)	0.42		1.01 (0.61~1.68)	0.96	
		Early preterm	Cluster 1 stable subgroup	0.79 (0.37~1.69)	0.54	0.94	1.19 (0.77~1.84)	0.42	0.96
			Cluster 2 shortened subgroup	0.82 (0.47~1.43)	0.48		1.17 (0.72~1.93)	0.52	
	5~8	Moderate to late preterm	Cluster 1 stable subgroup	1.08 (0.97~1.20)	0.15	0.81	1.00 (0.81~1.24)	0.98	0.03
			Cluster 2 shortened subgroup	1.05 (0.97~1.15)	0.23		**1.43 (1.08~1.89)**	**0.01**	
		Early preterm	Cluster 1 stable subgroup	**1.22 (1.01~1.46)**	**0.04**	0.26	0.70 (0.48~1.01)	0.06	<0.01
			Cluster 2 shortened subgroup	1.03 (0.85~1.24)	0.77		**1.62 (1.21~2.18)**	**<0.01**	
	9~12	Moderate to late preterm	Cluster 1 stable subgroup	0.96 (0.76~1.22)	0.76	0.29	0.76 (0.54~1.09)	0.13	0.42
			Cluster 2 shortened subgroup	1.12 (0.97~1.29)	0.12		0.87 (0.58~1.30)	0.50	
		Early preterm	Cluster 1 stable subgroup	0.73 (0.38~1.39)	0.33	0.95	0.96 (0.62~1.49)	0.86	0.41
			Cluster 2 shortened subgroup	0.71 (0.45~1.13)	0.15		1.21 (0.76~1.93)	0.42	
	13~16	Moderate to late preterm	Cluster 1 stable subgroup	0.96 (0.73~1.27)	0.79	0.66	0.84 (0.56~1.27)	0.42	0.13
			Cluster 2 shortened subgroup	0.91 (0.70~1.17)	0.45		1.43 (0.86~2.37)	0.17	
		Early preterm	Cluster 1 stable subgroup	1.28 (0.91~1.82)	0.16	0.29	1.00 (0.61~1.64)	1.00	0.07
			Cluster 2 shortened subgroup	0.99 (0.73~1.33)	0.92		**1.85 (1.03~2.98)**	**0.04**	
	17~20	Moderate to late preterm	Cluster 1 stable subgroup	1.09 (0.92~1.28)	0.32	0.78	1.13 (0.87~1.47)	0.37	0.46
			Cluster 2 shortened subgroup	1.06 (0.92~1.22)	0.43		1.34 (0.86~2.09)	0.20	
		Early preterm	Cluster 1 stable subgroup	1.21 (0.94~1.55)	0.14	0.37	0.70 (0.43~1.14)	0.15	<0.01
			Cluster 2 shortened subgroup	1.05 (0.83~1.32)	0.68		**1.65 (1.03~2.65)**	**0.04**	
	21~24	Moderate to late preterm	Cluster 1 stable subgroup	1.01 (0.85~1.20)	0.89	0.49	1.11 (0.93~1.33)	0.26	0.25
			Cluster 2 shortened subgroup	1.08 (0.96~1.22)	0.19		**1.38 (1.00~1.90)**	**0.05**	
		Early preterm	Cluster 1 stable subgroup	1.04 (0.78~1.40)	0.78	0.52	1.01 (0.72~1.42)	0.96	<0.01
			Cluster 2 shortened subgroup	**1.16 (1.01~1.33)**	**0.03**		**2.08 (1.48~2.94)**	**<0.01**	
	25~28	Moderate to late preterm	Cluster 1 stable subgroup	1.08 (0.94~1.24)	0.29	0.44	1.05 (0.84~1.31)	0.68	0.33
			Cluster 2 shortened subgroup	1.00 (0.88~1.13)	0.95		1.25 (0.93~1.67)	0.14	
		Early preterm	Cluster 1 stable subgroup	**1.21 (1.02~1.45)**	**0.03**	0.17	1.21 (0.97~1.52)	0.09	0.14

© 2024 Wu T et al. *JAMA Network Open*

			Cluster 2 shortened subgroup	**1.39 (1.26~1.52)**	**<0.01**		**1.58 (1.18~2.11)**	**<0.01**	
Lymphocyte-to-monocyte ratio	1~4	Moderate to late preterm	Cluster 1 stable subgroup	0.94 (0.70~1.27)	0.70	0.72	1.10 (0.76~1.59)	0.62	0.91
			Cluster 2 shortened subgroup	0.87 (0.68~1.10)	0.24		1.01 (0.63~1.61)	0.98	
		Early preterm	Cluster 1 stable subgroup	0.87 (0.44~1.70)	0.67	0.12	1.33 (0.86~2.06)	0.19	0.09
			Cluster 2 shortened subgroup	**1.47 (1.11~1.95)**	**<0.01**		0.76 (0.43~1.34)	0.34	
	5~8	Moderate to late preterm	Cluster 1 stable subgroup	0.93 (0.82~1.05)	0.22	0.56	0.94 (0.76~1.17)	0.60	0.04
			Cluster 2 shortened subgroup	**0.90 (0.82~0.99)**	**0.03**		**0.66 (0.49~0.90)**	**<0.01**	
		Early preterm	Cluster 1 stable subgroup	0.85 (0.62~1.18)	0.34	0.36	1.14 (0.86~1.51)	0.35	<0.01
			Cluster 2 shortened subgroup	0.99 (0.81~1.20)	0.88		**0.63 (0.45~0.89)**	**<0.01**	
	9~12	Moderate to late preterm	Cluster 1 stable subgroup	1.10 (0.89~1.37)	0.38	0.03	1.01 (0.76~1.33)	0.96	0.25
			Cluster 2 shortened subgroup	**0.79 (0.66~0.96)**	**0.02**		1.32 (0.91~1.92)	0.15	
		Early preterm	Cluster 1 stable subgroup	0.87 (0.53~1.44)	0.60	0.29	1.38 (0.94~2.04)	0.10	0.18
			Cluster 2 shortened subgroup	1.22 (0.90~1.65)	0.20		0.87 (0.51~1.48)	0.61	
	13~16	Moderate to late preterm	Cluster 1 stable subgroup	1.06 (0.83~1.37)	0.63	0.83	1.22 (0.85~1.76)	0.28	0.75
			Cluster 2 shortened subgroup	1.09 (0.89~1.33)	0.41		1.06 (0.66~1.71)	0.81	
		Early preterm	Cluster 1 stable subgroup	1.10 (0.70~1.71)	0.68	0.77	1.61 (0.99~2.61)	0.05	0.09
			Cluster 2 shortened subgroup	1.16 (0.87~1.55)	0.30		0.71 (0.40~1.26)	0.24	
	17~20	Moderate to late preterm	Cluster 1 stable subgroup	0.90 (0.74~1.09)	0.27	0.67	0.78 (0.57~1.06)	0.11	1.00
			Cluster 2 shortened subgroup	0.95 (0.81~1.10)	0.49		0.77 (0.50~1.20)	0.25	
		Early preterm	Cluster 1 stable subgroup	0.80 (0.52~1.25)	0.33	0.60	1.23 (0.85~1.78)	0.27	0.35
			Cluster 2 shortened subgroup	0.90 (0.70~1.16)	0.43		0.59 (0.35~1.01)	0.06	
	21~24	Moderate to late preterm	Cluster 1 stable subgroup	0.87 (0.72~1.05)	0.14	0.22	**0.72 (0.56~0.92)**	**<0.01**	0.69
			Cluster 2 shortened subgroup	**0.75 (0.64~0.88)**	**<0.01**		0.78 (0.58~1.05)	0.11	
		Early preterm	Cluster 1 stable subgroup	0.91 (0.63~1.30)	0.59	0.40	0.92 (0.67~1.26)	0.60	0.01
			Cluster 2 shortened subgroup	**0.74 (0.58~0.96)**	**0.02**		**0.52 (0.35~0.76)**	**<0.01**	
	25~28	Moderate to late preterm	Cluster 1 stable subgroup	0.90 (0.76~1.06)	0.20	1.00	0.82 (0.65~1.04)	0.10	0.86
			Cluster 2 shortened subgroup	0.90 (0.79~1.02)	0.10		0.79 (0.61~1.04)	0.09	
		Early preterm	Cluster 1 stable subgroup	0.77 (0.58~1.04)	0.09	0.58	0.90 (0.70~1.16)	0.42	0.55
			Cluster 2 shortened subgroup	**0.68 (0.57~ 0.81)**	**<0.01**		0.79 (0.60~ 1.04)	0.09	

© 2024 Wu T et al. *JAMA Network Open*
