# Supplementary material for: Longitudinal Cervical Length Measurements and Spontaneous Preterm Birth in Singleton and Twin Pregnancies
Source: JAMA Netw Open. 2024 Apr 11;7(4):e244592. doi: 10.1001/jamanetworkopen.2024.4592 (PMC11009824; doi:10.1001/jamanetworkopen.2024.4592)
Supplement: Supplement 2. — Data Sharing Statement [file jamanetwopen-e244592-s002.pdf]

Data Sharing Statement

Wu. Longitudinal Cervical Length Measurements and Spontaneous Preterm Birth in Singleton and Twin Pregnancies. *JAMA Netw Open*. Published April 11, 2024.
doi:10.1001/jamanetworkopen.2024.4592

Data
Data available: No
